# Structure of the Hexadecane Rotator Phase: Combination of X-ray Spectra and Molecular Dynamics Simulation

**DOI:** 10.1021/acs.jpcb.3c02027

**Published:** 2023-08-30

**Authors:** Stephen A. Burrows, E. Emily Lin, Diana Cholakova, Sam Richardson, Stoyan K. Smoukov

**Affiliations:** † Centre for Sustainable Engineering, School of Engineering and Materials Science, Queen Mary University of London, Mile End Road, London E1 4NS, U.K.; ‡ Department of Chemical and Pharmaceutical Engineering, Faculty of Chemistry and Pharmacy, Sofia University, Sofia 1164, Bulgaria

## Abstract

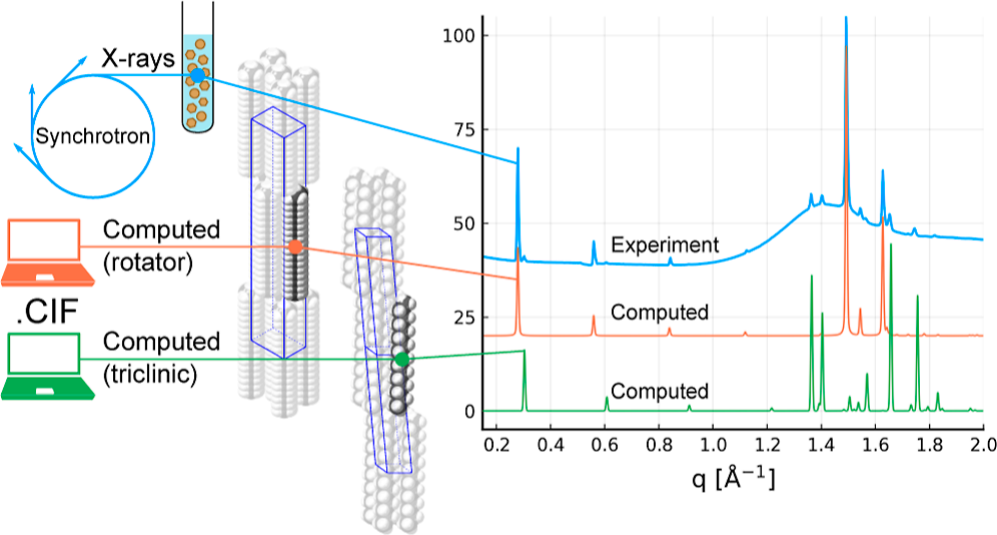

Rotator phases are rotationally disordered plastic crystals, some of which can form upon freezing of alkane at alkane–water interfaces. Existing X-ray diffraction studies show only partial unit cell information for rotator phases of some alkanes. This includes the rotator phase of *n*-hexadecane, which is a transient metastable phase in pure alkane systems, but shows remarkable stability at interfaces when mediated by a surfactant. Here, we combine synchrotron X-ray diffraction data and molecular dynamics (MD) simulations, reporting clear evidence of the face-centered orthorhombic R_I_ rotator phase from spectra for two hexadecane emulsions, one stabilized by Brij C10 and another by Tween 40 surfactants. MD simulations of pure hexadecane use the recently developed Williams 7B force field, which is capable of reproducing crystal-to-rotator phase transitions, and it also predicts the crystal structure of the R_I_ phase. Full unit cell information is obtained by combining unit cell dimensions from synchrotron data and molecular orientations from MD simulations. A unit cell model of the R_I_ phase is produced in the crystallographic information file (CIF) format, with each molecule represented by a superposition of four rotational positions, each with 25% occupancy. Powder diffraction spectra computed using this model are in good agreement with the experimental spectra.

## Introduction

Artificial morphogenesis is a recently discovered process where alkane emulsion droplets can transform into a variety of geometric shapes upon cooling when stabilized by certain surfactants.^1−5^ Many of these shapes, such as hexagonal platelets, have significantly higher surface area than a sphere of the same volume and require elastic forces to counteract the surface tension, which would otherwise keep the droplets spherical.^6,7^ Partial crystallization of the droplet generates enough solid material to produce the necessary elastic stress, and though the molecular mechanism is still not completely known, there are a number of applications that have emerged for this process. Cooled emulsions have been found to self-emulsify through several mechanisms associated with the droplet’s shape transformations.^8,9^ Using an ultraviolet (UV)-initiated polymerizable monomer in the oil phase, highly shape-anisotropic solid nanoparticles have been produced.^10^

*n*-Hexadecane (abbreviated to C_16_ or C_*n*_ for other *n*-alkanes) is the oil phase most widely used to demonstrate this shape-changing behavior with a variety of surfactants, although many other alkanes and similarly long molecules exhibit the phenomenon too.^11^ C_16_’s chain of 16 saturated carbon atoms is characteristic of the alkyl group in many important biomolecules including palmitic acid, triglycerides, and phospholipids. It conveniently has a melting point close to room temperature of 291 K or 18 °C, so the working temperature range to achieve phase transitions is close to ambient conditions (5–20 °C, accounting for supercooling and full melting).

An interesting property of artificial morphogenesis is that while the interior of the droplet stays liquid, the C_16_ solidifying at the interface often exhibits a crystal structure different from the lowest energy triclinic phase. Instead, it forms an orthorhombic “rotator” phase.^12,13^ In this lower-density structure, the molecules have the freedom to rotate about their long axis but may still have preferred orientations which depend on the specific rotator phase. The rotator phase of C_16_ is not stable in pure alkane but has been found to play a unique role in these shape transformations.^14^ It is known to form layers of varying thickness at the alkane–water interface which, while strong enough to counteract the interfacial tension, are soft and deformable plastically, allowing shape rearrangement. Evidence from differential scanning calorimetry experiments suggests that the rotator phase may reach tens of layers in thickness while the shape transformations are ongoing.^15^ An alternative mechanism has been proposed, in which interfacial tension can become sufficiently low during cooling (ca. 0.1 mN/m) that a monolayer is capable of deforming the droplets.^3,4^

The most stable crystalline form of C_16_ has a triclinic unit cell (*P*1̅ space group) containing a single molecule. This space group has a single point of inversion coinciding with the point symmetry of C_16_. Its lattice dimensions and atomic coordinates were determined by Métivaud et al.^16^ and uploaded to the Cambridge Crystallographic Data Centre (CCDC) database.^17^ Odd and even *n*-alkanes may have different crystal structures even if there is only one carbon atom difference. For example, C_15_ has an orthorhombic unit cell, whereas C_16_ has a triclinic one. Another difference is that the rotator phase of C_15_, known as the R_I_ phase, is stable over a temperature range of approximately 10 K upon both cooling and heating, whereas the rotator phase of C_16_ is only observed for several seconds upon cooling in bulk or for a longer period in the presence of interfaces.^12−14^

**Figure 1:**
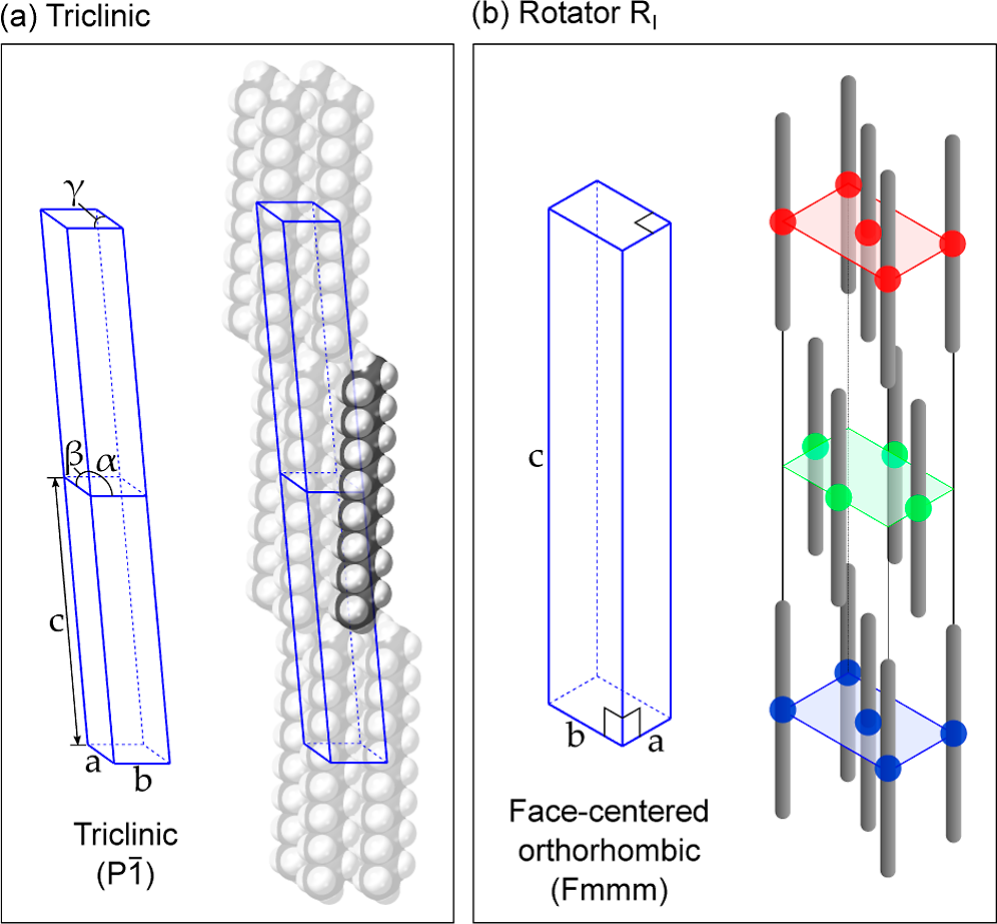
(a) Unit cell and structure of the triclinic crystal phase; (b) unit cell and structure of orthorhombic rotator phase R_I_. Two unit cells are shown for the triclinic phase to provide a direct comparison to the rotator phase, which has a height equal to two lamellar layers. Rotator phase molecules are drawn as rods to illustrate the rotational disorder.

Ungar^12^ determined the space group of the R_I_ unit cell for the odd *n*-alkanes C_11_ to C_25_. After establishing that the X-ray diffraction peaks were consistent with an orthorhombic unit cell, the reflection conditions and symmetry of the molecule were sufficient to determine that the rotator phase has the *Fmmm* space group. For this structure, the height of the unit cell along the crystallographic *c* axis corresponds to two lamellar layers and it is the corresponding [002] peak which first appears in small-angle X-ray scattering (SAXS) spectra. The symmetry elements of this space group may seem incompatible with C_16_, such as a mirror plane bisecting the molecule with its normal parallel to the *c* lattice vector, considering that C_16_ does not have such mirror symmetry. However, the rotator phase symmetry corresponds to a time-averaged structure in which the different possible orientations of the molecule are superposed, allowing even numbered *n*-alkanes such as C_20_ to also demonstrate the R_I_ phase.^18^ The contrasting structures of the triclinic crystal and orthorhombic rotator phases are visualized in Figure 1. At higher temperatures, a hexagonal rotator phase (R_II_) can form. It is unlikely that the R_II_ phase could be observed experimentally for C_16_ as it has only been observed for longer alkanes at temperatures above the C_16_ melting point. The temperature dependence of the rotator phase lattice parameters was also reported by Ungar for C_11_ to C_25_.^12^

Sirota and Herhold^13^ observed the rotator phase using synchrotron X-ray diffraction experiments in pure even *n*-alkanes C_16_ to C_22_ by crystallizing them from the supercooled liquid. The stability of the rotator phase increases with chain length, so the rotator phase of C_16_ was observed only for a short time during approximately 10% of the repeated experiments. It was observed only upon cooling from the liquid, whereas C_22_ exhibited the rotator phase upon heating and cooling. Sirota described the rotator phase of C_16_ as “transient”, implying a short lifetime metastable phase. Di Giambattista et al.^18^ investigated crystal lattice relaxations of the rotator phase of C_20_ under temperature jumps using calorimetry and X-ray diffraction. They found that the relaxation rates of the *a* and *b* lattice parameters were significantly different. They also showed that the relaxation times increased with temperature up until the melting point, in contrast to classical thermally activated processes.

The rotator phase of C_16_ has been more consistently observed at interfaces. This can be achieved by creating an emulsion of liquid oil droplets, typically 1−100 microns in size, suspended in water. The system is cooled sufficiently to induce crystallization of the alkane, which is known to begin at the interface and can be mediated by the alkyl tails of the surfactant.^2−4,14,15^ The temperature remains above 0 °C, so the water remains liquid at all times. Emulsion experiments by Ueno et al.^19^ and Shinohara et al.^20^ detected X-ray diffraction peaks from C_16_ droplets consistent with an orthorhombic unit cell, in addition to the lowest energy triclinic phase. A recent study by Cholakova et al.^14^ suggests that even two different rotator phases of C_16_ may occur during the transition from the liquid to triclinic phases, depending on the surfactant and droplet size. Rotationally disordered phases have also been observed in other molecules, such as *n*-alkyl trimethylammonium bromide surfactants.^21^

There are also examples of simple multi-component fluid systems where the presence of an interface leads to stability of a condensed phase, localized to the interface, which would not be stable in the equivalent homogeneous system of that component. For example, gaseous CO_2_ may adsorb to form a thin liquid layer, which wets the interface between the decane + CO_2_ solution and the CO_2_ vapor.^22^ The same effect has also been observed in two-component Lennard-Jones fluid systems, where the supercritical component can wet the interface between the subcritical component (liquid) and the vapor.^23^

Only partial information is available for unit cell parameters of alkane rotator phases, and crystallographic information files (CIFs) are not present in existing databases. Furthermore, X-ray spectra are sufficient to determine the space group, but not necessarily able to determine the distribution of molecule orientations at the different molecular locations as many possible distributions can be consistent with a single space group.

Unsurprisingly, a large number of molecular dynamics (MD) force fields for alkanes are available with a wide range of potential energy functions and levels of coarse graining, as well as lipid force fields which can be readily adapted. Their performance has been reviewed for reproducing different alkane properties such as liquid phase transport^24,25^ or phase transition temperatures and solid phase ordering.^26,27^ Here, we focus on studies looking specifically at modeling of rotator phases for higher alkanes.

Early attempts to model alkane rotator phases using MD were performed by Ryckaert and Klein using an infinite-chain approximation.^28^ They observed the characteristic rotational disorder of the molecules at high temperatures (375 K) but without the discontinuous jump in lattice parameters expected of the first-order crystal-to-rotator transition. Marbeuf and Brown were able to simulate the R_I_ phase of alkanes C_18_–C_20_ using the COMPASS force field,^29^ which uses a 9-6 Lennard–Jones potential for intermolecular interactions.^30^ Further research by Wentzel and Milner^31,32^ showed that force fields based on the Williams Buckingham potential^33^ could reproduce the correct sequence of solid phases upon heating, including two rotator phases, for the odd *n*-alkanes C_21_ and C_23_. Recently, the Williams force field has been optimized to accurately reproduce melting points and transport properties in addition to crystal-rotator phase transitions.^27^

Iliev et al.^34^ utilized long simulation times of up to 500 ns to study nucleation and crystallization of C_16_ from the liquid, both in bulk and at the water/C_16_ interface with surfactant, using the CHARMM36 force field. This resulted in systems containing many small crystallites, with the tilt angle of the molecules (relative to the crystallite plane) varying considerably between them. Orientational disorder characteristic of the rotator phase was seen transiently for many crystallites, and the largest crystallite ultimately settled into an ordered structure resembling the triclinic phase.

**Figure 2:**
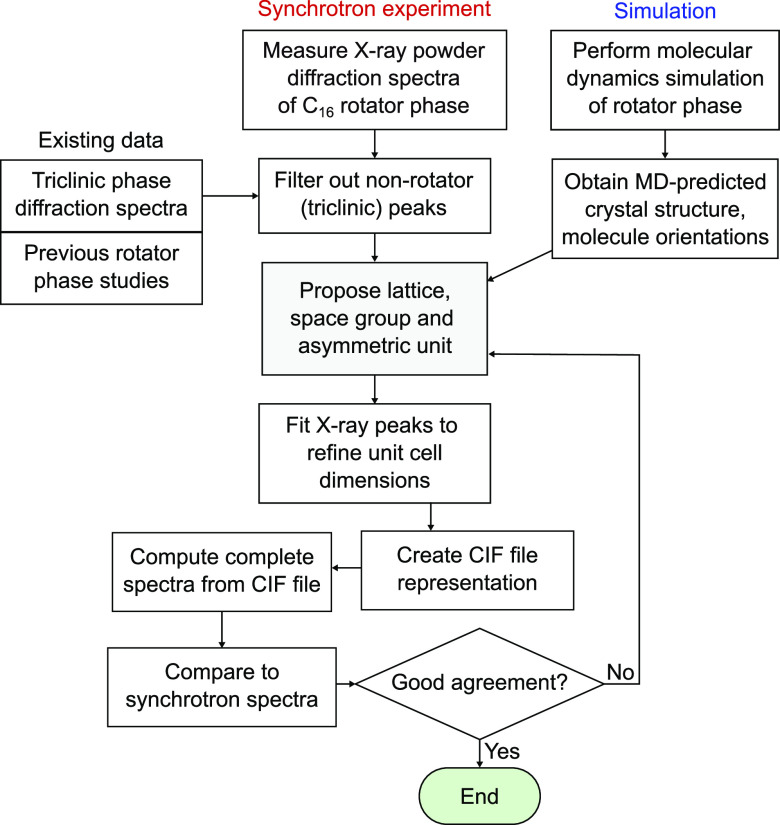
Flowchart illustrating the overall strategy to create a realistic CIF of the C_16_ rotator phase, using experiment and simulation data.

In this work, we combine synchrotron X-ray diffraction spectra with information from MD simulations to create a unit cell of the C_16_ rotator phase. We use the X-ray diffraction data to establish the stability of the rotator phase of C_16_ in surfactant-stabilized emulsions, identifying and indexing the most prominent diffraction peaks. Then, we perform MD simulations of pure C_16_ using the recently optimized Williams 7B alkane force field,^27^ which can reproduce crystal-to-rotator phase transitions. We analyze the positional and rotational order of the C_16_ molecules and compare them to the experimentally determined lattice parameters and symmetries. Extracting unit cell dimensions from the X-ray spectra and molecular orientations from the MD simulations, we produce a unit cell geometry file in the CIF format. Finally, we use this to compute the full powder diffraction spectra of the rotator phase to validate our results. This strategy is illustrated by the flowchart in Figure 2.

## Methods

### Experimental Methods

#### Materials and Emulsion Preparation

Polyoxyethylene alkyl ether (C_16_EO_10_, Brij C10) and polyoxyethylene sorbitan monoalkylate (C_16_SorbEO_20_, Tween 40) non-ionic surfactants and *n*-hexadecane (C_16_) were purchased from Sigma-Aldrich. All the materials were used as received.

C_16_ emulsions stabilized by Brij C10 or Tween 40 were prepared by membrane emulsification with a Shirasu porous glass membrane. Brij C10 stabilized emulsions were prepared using a 1 wt % Brij C10 solution in water and 10 μm pore-size membrane, resulting in ca. 33 μm droplets. Tween 40-stabilized emulsions were prepared using a 1.5 wt % Tween 40 solution in water and a 3 μm pore-size membrane, resulting in ca. 13 μm droplets.

Cooling of the emulsions was realized in round capillaries positioned within the sample holder, so the X-ray beam passes through the top of the sample where buoyant droplets may accumulate. The chamber temperature was controlled by using a cryothermostat and measured close to the emulsion location, using a calibrated thermocouple probe with an accuracy of ±0.2 °C.

Experiments began at ambient conditions of ca. 21 °C (294 K), which is just above the C_16_ melting point of 18 °C (291 K), and therefore, oil droplets were entirely in the liquid phase. The temperature profile for each experiment is plotted as an inlay on the corresponding spectra figure.

#### Synchrotron Facility

Experiments were performed at the SOLEIL national synchrotron facility in France, on the SWING beamline^35^ using 16 keV radiation. An EIGER-4M detector with an active area of 162.5 × 155.2 mm was used, and the distance from the sample to the detector was 472 mm. This allowed a measurable *q* range of 0.026 to 3.398 Å^–1^, where *q* is the scattering vector magnitude and is related to the interplanar spacing *d* by *q* = 2π/*d*. Diffraction intensity was recorded as a function of *q* with a resolution of 0.0025 Å^–1^.

#### Spectral Peak Fitting

To extract lattice dimensions from the X-ray diffraction spectra, the precise location of the diffraction peaks must be determined. First, a cubic polynomial is fit to the background spectra by selecting a part of the surrounding spectra, either side of the target peak(s), where no other peaks are present. This region chosen for background fitting will be marked on the corresponding peak fitting figures. Once the background is established and subtracted, each peak is initially fit using Lorentzian and Gaussian functions separately. All fitting is performed using the LsqFit package of the Julia programming language.^36^ Having determined the optimum values for the Lorentzian and Gaussian parameters, a weighted sum of the two functions is fit to the peak to obtain a pseudo-Voigt profile
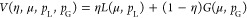
1where η determines the weighting of the Lorentzian and Gaussian functions, which are denoted *L* and *G*, respectively, μ is the location of the peak, and *p*_L_ and *p*_G_ are the remaining parameters of the Lorentzian and Gaussian functions, respectively. This achieves a better fit to the peak shape and provides information on the Lorentzian–Gaussian parameter, η, with the convention that η = 1 denotes fully Lorentzian, as adopted by CrystalDiffract.^37^

If the peak of interest overlaps with other peaks, then a sum of two or more Lorentzian/Gaussian functions is used, each of which has its own independent parameters which are optimized. In the case of two overlapping peaks, the combined (pseudo-Voigt) function becomes
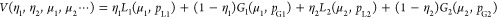
2where subscript 1 denotes the first peak and subscript 2 denotes the second peak. Therefore, each peak has its own location μ and Lorentzian–Gaussian parameter η.

### Simulation Methods

#### MD Force Field and Settings

In this work, we use the recently optimized Williams 7B alkane force field, which has shown the ability to reproduce the crystal-rotator phase transition of C_15_.^27^ Williams 7B is an all-atom force field, which uses a Buckingham potential to model non-bonded interatomic interactions, where the pairwise energy for two atoms separated by a distance *r* is given by
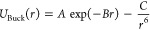
3

The original Williams force field was optimized to reproduce crystal structures, heats of sublimation, and elastic moduli of hydrocarbons.^33^ Williams 7B was created by modifying the Buckingham potential well depth, while maintaining its shape at the energy minima, in order to correctly reproduce transport properties when using a pair potential cut-off of 10 Å.^27^ The optimized force field was also shown to reproduce the melting point of C_15_/C_16_ and the crystal-rotator phase transition temperature of C_15_ more accurately.

Parameters for the Williams 7B force field including bond, angle, and dihedral terms are provided in Supporting Information Tables S1 and S2, with details on parameterization given in ref (27). Here, C–H bond lengths are not constrained, instead using the harmonic bond coefficient in Table S2. In ref (27), this force field was implemented by tabulating the pair potential because this was more computationally efficient for Buckingham potentials in GROMACS. Here, we use the LAMMPS MD software^38^ (version 29, Oct 2020), which has an optimized implementation of Buckingham potentials for Intel CPUs and other architectures, meaning that no tabulation is necessary. Force field parameters in the Moltemplate format^39^ are provided in the Supporting Information to assist in preparing LAMMPS input files using the Williams 7B force field.

The Buckingham potential was truncated at 10 Å using a plain cut-off, as was used in the Williams 7B optimization. The long-range tail correction to energy and pressure was applied, by specifying “pair_modify tail yes” within the LAMMPS input file. Equations of motion were integrated using the LAMMPS velocity–Verlet integrator and a time step of 1 fs for all simulations. Thermodynamic information and system dimensions (which are used to extract the lattice parameters) were saved every 100 time steps (0.1 ps), and coordinates were saved every 100 ps.

All simulations were performed at constant temperature and pressure (NPT ensemble). The LAMMPS fully-anisotropic barostat, using the Parrinello–Rahman expression for shear strain energy,^40^ was used to control the pressure. In our previous work,^27^ we found that simulations of alkane crystals using an undamped Parrinello–Rahman barostat have resulted in large-amplitude oscillations of the system dimensions. Therefore, we enable the drag term in the barostat, with a drag factor of 0.2—the lower end of the 0.2–2.0 range suggested in the LAMMPS documentation. In all cases, the diagonal components of the pressure tensor were fixed to 1 bar and the off-diagonal components (shear stresses) to zero. The temperature was controlled using a Nosé–Hoover thermostat with a time constant *t*_damp_ = 0.1 ps.

To measure the lattice parameters of the triclinic phase, simulations of 40 ns in length were performed at 273, 278, 283, and 288 K, and the dimensions of the unit cell were averaged over this time. Reported triclinic lattice parameters correspond to the standard primitive unit cell^16^ which contains one molecule.

To simulate the rotator phase, the triclinic crystal was heated until a solid–solid phase transition occurs, after which the molecules were no longer tilted with respect to the layer normal. It was found that heating from 305 to 315 K over a 10 ns period (1 K/ns heating rate) was sufficient for this transition to occur. Then, the system was cooled down from 315 K to the desired measurement temperature, also at a rate of 1 K/ns. 10 ns of constant temperature NPT equilibration was then performed before starting data production simulations. 40 ns simulations were used to compute lattice parameters of the R_I_ phase at temperatures of 288, 293, 298, and 303 K. These temperatures include some which are higher than the melting point of C_16_. However, superheating of crystals is possible in MD simulations due to the energy barrier required to form a liquid nucleus inside an infinite crystal, which completely spans the periodic simulation box.

#### Preparing Initial Configuration

The starting configuration for our MD study is a supercell of pure C_16_ in its stable triclinic structure. The triclinic unit cell of C_16_ determined by Métivaud et al.^16^ was used to define the initial atomic positions. The corresponding CIF was downloaded from the CCDC database.^17^ Before building a supercell using this structure, the unit cell was transformed into a non-primitive unit cell as described below.

MD simulation programs such as LAMMPS and GROMACS have certain criteria, which must be fulfilled for non-orthogonal simulation boxes. For example, if the vectors , , and  define the edges of the periodic simulation box, then the constraint  must be satisfied, where the subscript *x* denotes the *x* component. This is problematic if the triclinic cell of C_16_ transitions to a hexagonal rotator phase in which γ (the angle between  and ) approaches 60° as this constraint may be violated. Therefore, the triclinic unit cell of C_16_ is transformed into a non-primitive unit cell, where the angles between the unit cell vectors will be close to 90° if the triclinic-to-rotator transition occurs. The transformed cell has lattice vectors , , and , where  and . The vector  is unchanged by the transformation, hence . A visual representation of this transformation is given in Supporting Information Figure S1. This transformed unit cell has 4 times the volume of the original triclinic cell and contains four molecules.

At the temperature range used in the synchrotron experiment, the rotator phase we most expect to see is the R_I_ phase. This phase has an ABAB layer stacking sequence, which means an even number of layers in the periodic simulation box are needed to allow this phase to appear. However, rotator phases with ABCABC stacking have also been observed.^12^ Therefore, six layers are used as this is divisible by both 2 and 3, allowing AB and ABC stacking to develop.

Using the transformed unit cell, a supercell consisting of 20 × 10 × 3 unit cells in the *a*, *b,* and *c* lattice directions, respectively, was chosen. This corresponds to the desired six lamellar layers because each unit cell contains two layers along the *c* lattice direction. The total number of molecules in this supercell is 2400.

#### Obtaining Rotator Phase Structure from Simulations

To determine the crystal structure of the rotator phase in the MD simulations, we start by studying the positional order of the molecules. The crystal is divided into its lamellar layers, and the spatial density profile is computed for each layer by computing a histogram of molecule positions. The center of mass of each molecule is calculated at each frame, and these are converted to fractional coordinates. The three vectors defining the orthorhombic unit cell are used as the basis vectors for the fractional coordinates. As the rotator phase is orthorhombic, these unit cell vectors can simply be obtained by dividing the simulation box vectors by the number of unit cells in each dimension. The benefit of the fractional coordinates is that the molecule positions are defined with respect to the lattice points, hence elastic deformation/vibration of the simulation box does not blur the density profile. Density profiles are computed in the *ab*-plane, and the profiles of three subsequent layers are assigned to the color channels of an RGB image, which allows us to visualize the density of up to three layers at once. If two layers are directly on top of each other, their color channels will add together (i.e., red and blue will sum to magenta).

A useful parameter to differentiate the orthorhombic R_I_ and hexagonal R_II_ phases is the ratio of lattice parameters *b* to *a*, referred to as the *b*/*a* ratio. If we assign the lattice vectors using the convention that *a* is smaller than *b*, the geometry of the hexagonal R_II_ phase requires that *b*/*a* is exactly equal to , whereas for the R_I_ phase *b*/*a* is , temperature-dependent, and approximately 1.5 at room temperature.

**Figure 3:**
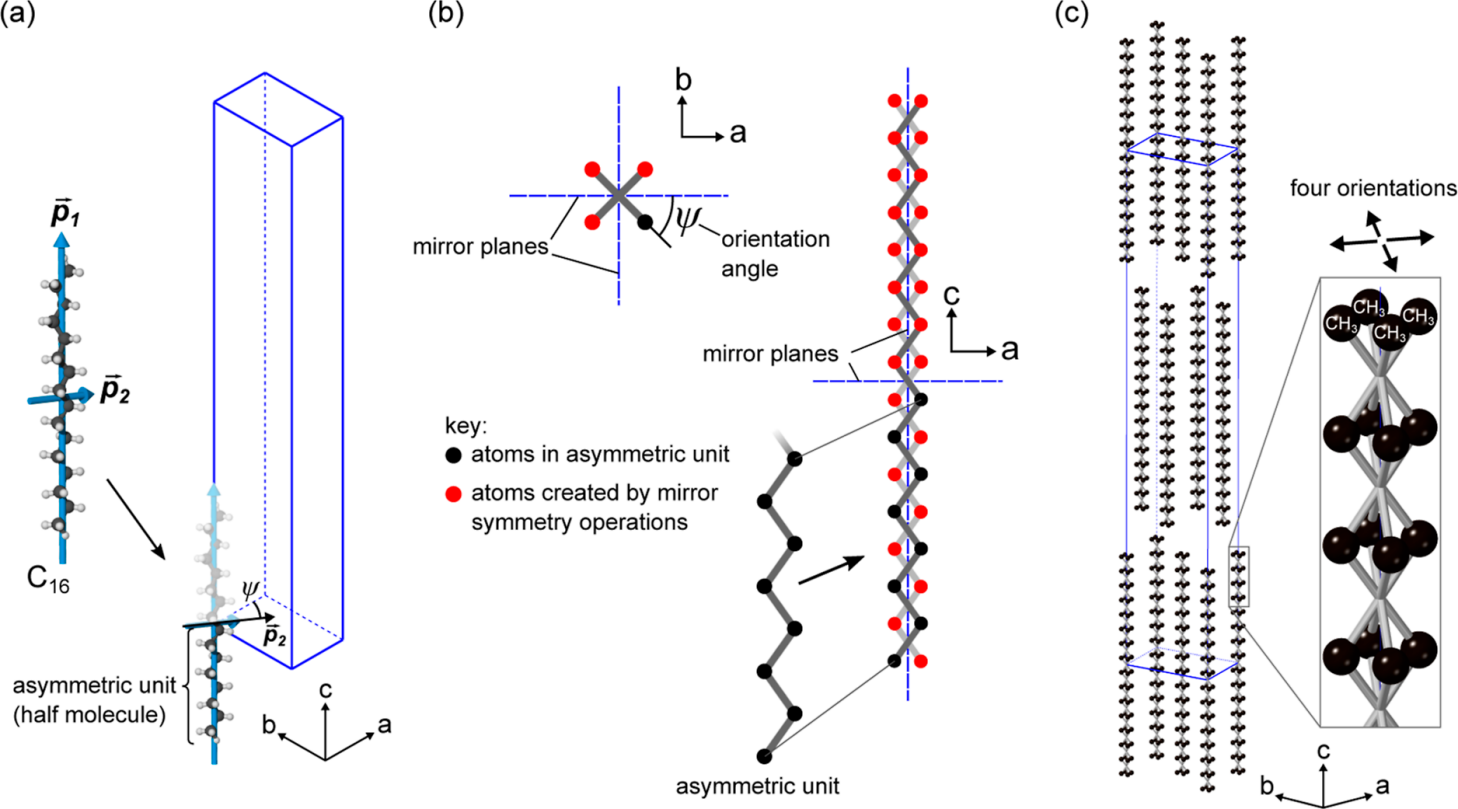
(a) Principal component vectors  and  define the orientation of the molecule; (b) application of *Fmmm* symmetry elements (mirror planes) creates a superposition of four possible orientations; (c) final face-centered orthorhombic cell and visualization of the four resulting orientations (H atoms hidden for clarity).

To quantify the level of rotational disorder, we start by computing the orientation of each molecule using the principal component method used in our previous work.^27^ Each molecule has an orientation angle ψ in the *ab*-plane, defined as the angle between the second principal component vector  and the *a* axis, which measures rotation about the long axis  of the molecule as shown in Figure 3a. A histogram of these orientation angles is produced with a bin size of 5°, which is normalized to create a discrete probability distribution *P*(ψ). The normalized entropy of this distribution is given by
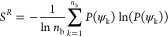
4where *n*_b_ is the number of discrete values of ψ (*n*_b_ = 72 when using 5° bin size). *S*^*R*^ can then be used to quantify the level of rotational disorder as in ref (27). A value of unity corresponds to the case where all orientations are equally probable, i.e., complete rotational disorder, which is characteristic of the R_II_ phase.

### Constructing the Rotator Phase Unit Cell

To the best of our knowledge, a full structural model of the C_16_ rotator phase R_I_ unit cell has not been created previously. Therefore, we construct one in the CIF format, which can be used to compute idealized X-ray powder diffraction spectra for direct comparison to the experiment.

Previous X-ray diffraction studies have reported three particularly intense peaks for the rotator phase. These are [002] which corresponds to the lamellar layer spacing *d*_002_, followed by [111] and [020]. The peaks [001] and [010] are systematic absences for the face-centered orthorhombic lattice. The [111] peak is particularly intense due to having a reflection multiplicity of 8, i.e., there are eight symmetry-equivalent reflections contributing to the intensity of a single diffraction peak. From these three peaks, the dimensions of the unit cell (*a*, *b*, and *c*) can be calculated if it is assumed the unit cell is orthorhombic, which the R_I_ phase is known to be. First, *b* and *c* are simply computed as *b* = *d*_010_ = 2*d*_020_ and *c* = *d*_001_ = 2*d*_002_, respectively. Then, *a* is computed using the general formula for interplanar spacing, *d*_*hkl*_, for orthorhombic lattices
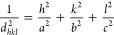
5where *hkl* are the Miller indices. In our case, *d*_111_ is known from the spectra, so this can be rearranged to compute *a* as
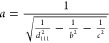
6

After obtaining these dimensions, an atomistic model of the rotator phase unit cell is constructed and a CIF is produced. To do this, the space group and the fractional coordinates of atoms in the asymmetric unit must be specified in addition to the unit cell dimensions. Within the CIF, the C_16_ molecule is in the straight all-trans conformation, known to be the dominant conformer in the crystal and rotator phases of *n*-alkanes.^41−43^ The internal geometry, i.e., bond lengths and valence angles, are not modified from the triclinic phase structure determined by Métivaud et al.^16^ The Cartesian coordinates are determined by translating and rotating the C_16_ molecule so that its center of mass and orientation are consistent with the crystal structure determined from the spectra and simulation analysis.

Here, we consider the case where the space group is *Fmmm*, as expected for the R_I_ phase. In this case, the molecule’s center of mass will lie at the origin of the face-centered orthorhombic unit cell. Then, the Cartesian coordinates of one-half of the molecule (corresponding to the asymmetric unit), that is carbons 1 to 8 including attached hydrogens, are converted to fractional coordinates and recorded in the CIF.

The placement of the molecule in the *Fmmm* unit cell is shown in Figure 3. We see why the asymmetric unit only contains half the molecule as applying the mirror symmetry operations generates all other atom positions. The resultant structure contains a superposition of four orientations at each lattice point, all of which are vertical ( parallel to *c*), but with the  vector at ±ψ and ±(180° – ψ). The atom entries in the CIF are therefore assigned an occupancy of 0.25 as they correspond to one of four equally probable orientations. In Figure 3b, the four orientations can be seen in a top-down view of the *ab*-plane.

#### Computing Spectra from the CIF

After producing a CIF representation of the R_I_ phase unit cell, the CrystalDiffract software^37^ was used to generate a powder diffraction pattern. CrystalDiffract allows the Lorentzian–Gaussian parameter η (eq 1) to be adjusted in order to achieve the most representative peak shape. This was set equal to the average value of η for the X-ray diffraction peaks, as determined from the peak fitting procedure outlined above. This was found to be η = 0.4, implying peaks slightly closer to Gaussian in shape.

## Results and Discussion

### Experimental Results

#### X-ray Diffraction Spectra

**Figure 4:**
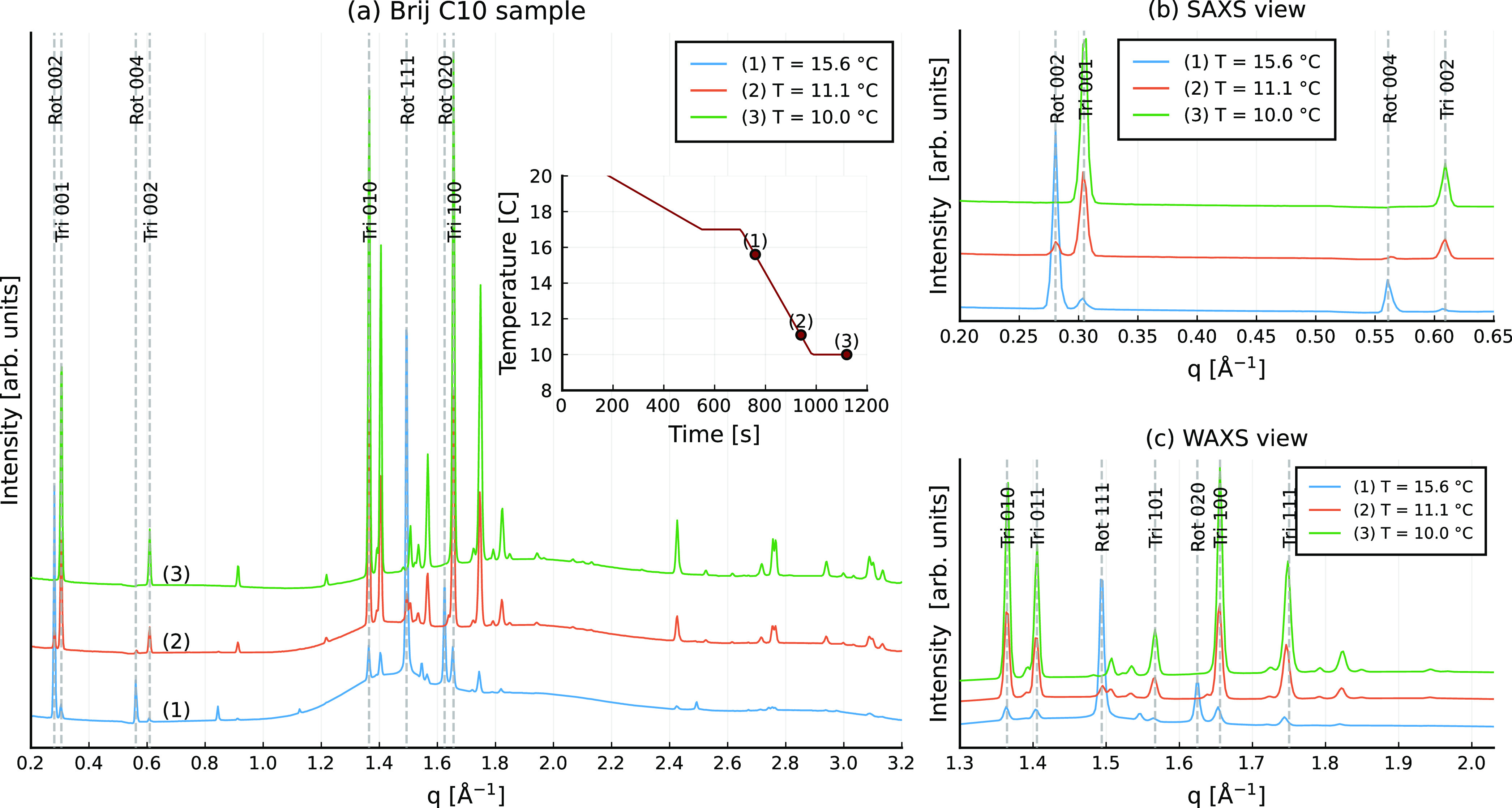
X-ray diffraction pattern obtained from the Brij C10-stabilized C_16_ emulsion. Peaks are labeled with “rot” or “tri” for rotator and triclinic phases, respectively, followed by the three Miller indices; (a) full spectra and the temperature profile (inlay); (b) close-up of SAXS peaks; (c) close-up of WAXS peaks.

**Figure 5:**
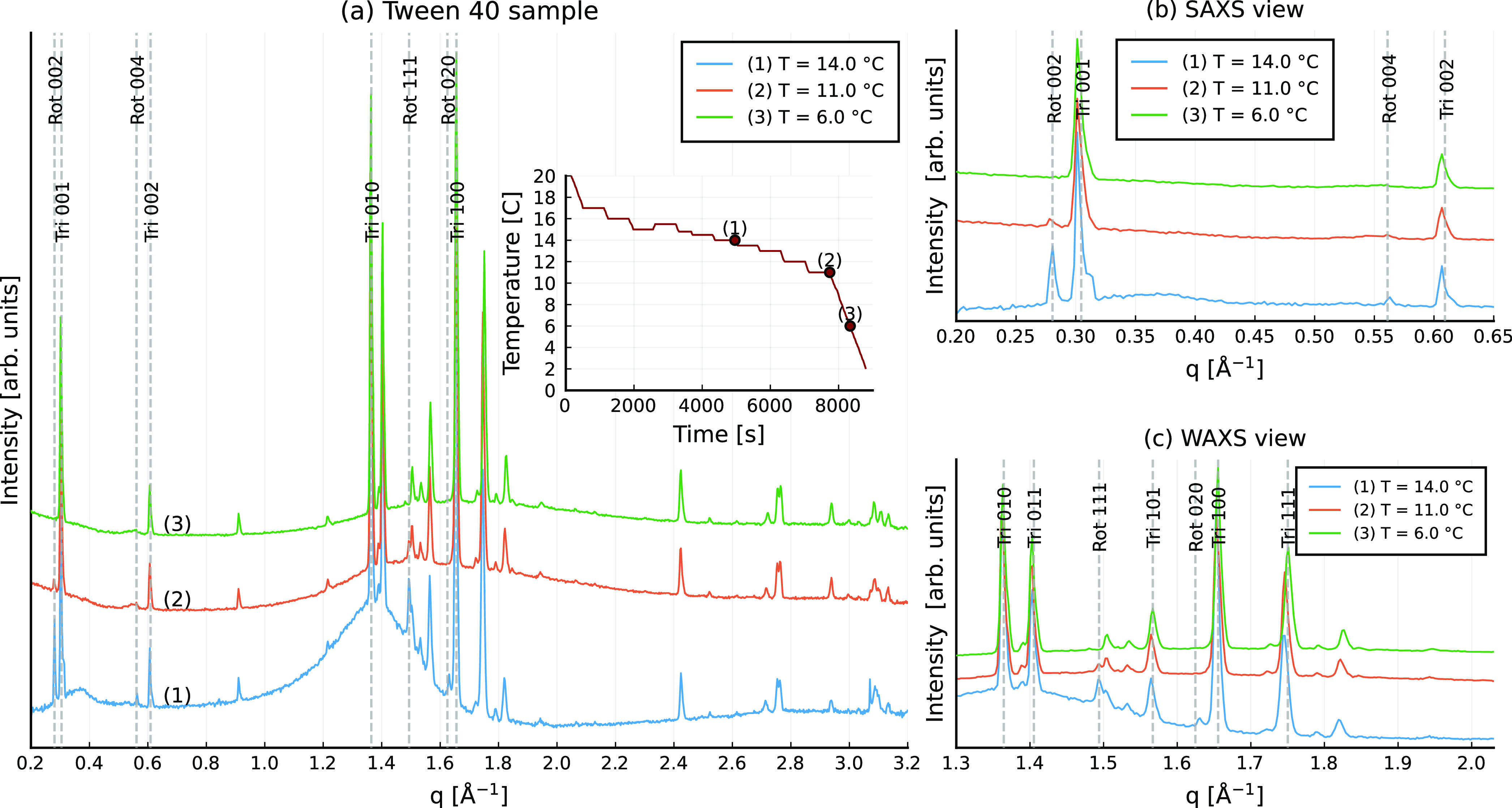
X-ray diffraction pattern obtained from the Tween 40-stabilized C_16_ emulsion. Peaks are labeled with “rot” or “tri” for rotator and triclinic phases, respectively, followed by the three Miller indices; (a) full spectra and the temperature profile (inlay); (b) close-up of SAXS peaks; (c) close-up of WAXS peaks.

Diffraction patterns obtained using synchrotron X-ray diffraction are shown in Figures 4 and 5 for the Brij C10 and Tween 40 stabilized emulsions, respectively. Expected locations of the strongest peaks for the triclinic and rotator (R_I_) phases are shown to help indicate which phases are present. The three most intense rotator phase peaks ([002], [111], and [020]) were observed in all samples upon cooling prior to the formation of the triclinic crystalline phase. Usually, the rotator phase occurred at a temperature of 15.5–16 °C and remained present in the sample down to 8–9 °C. The triclinic phase becomes dominant around 11 °C for both emulsions. Upon further cooling or if the temperature was kept low for a prolonged period of time, all hexadecane molecules arrange in the triclinic phase, and the rotator phase disappears.

The amount of rotator phase formed in Brij C10 stabilized emulsions was much larger compared to that found in Tween 40 stabilized emulsions. This is consistent with the analysis in ref (14), where it is shown that all alkane molecules are arranged in the R_I_ phase before their full crystallization for the Brij C10 stabilized systems (see Figure 7c in ref (14)). In contrast, in Tween 40-stabilized emulsions, the rotator phase only remains present near the surface of the droplets, while the interior of the deformed particle undergoes a direct liquid-to-crystal (triclinic) phase transition.

#### X-ray Peak Fitting

The Brij C10-stabilized emulsion spectra were used for further analysis because they contained rotator phase peaks that were significantly stronger than the triclinic ones. The spectrum measured at 15.6 °C was used to fit peak locations for the rotator phase. Six peaks were identified by comparison to the expected *d*-spacings of the detected orthorhombic R_I_ phase, which were [002] (which corresponds to the spacing between lamellar layers), [111], [113], [020], [200], and [131]. *q* values for three of these peaks ([113], [200], and [131]) were not previously reported. This is due to the weak intensity of these peaks, and in the case of [200] and [131], being outside previously reported *q* ranges.^13,14,19,20^

**Figure 6:**
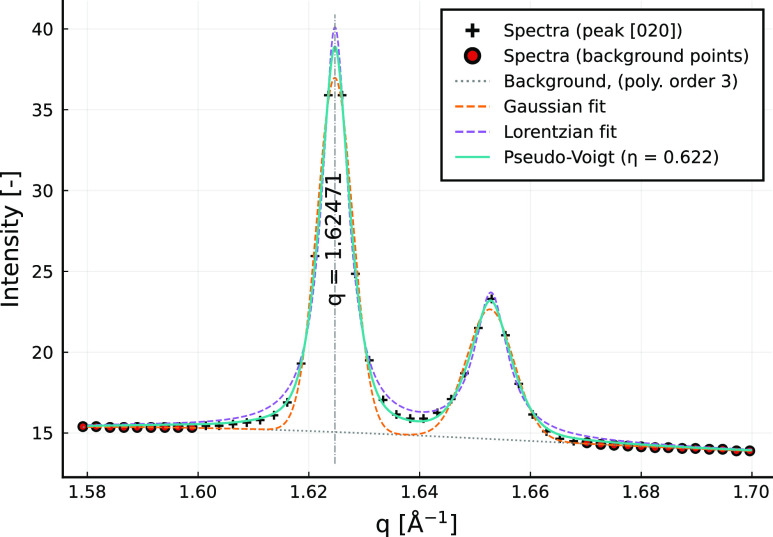
Results for peak fitting of the rotator phase [020] peak. This peak is chosen for visualization due to the presence of a neighboring peak (right side), which is the [100] peak of the triclinic phase which is also present.

Figure 6 shows the fitting of the [020] R_I_ phase peak, occurring at *q* = 1.625 Å^–1^. It is seen that the pseudo-Voigt function offers a better quality of fit than the Gaussian or Lorentzian functions alone, in this case with η ≈ 0.6. We also find that the peak fitting is still successful when the neighboring triclinic [100] peak is overlapping. Equivalent figures for the remaining peaks ([002], [111], [113], [200], and [131]) are provided in Supporting Information Figures S2–S6. The *q* values of the six identified peaks are given in Table 1. We find that the locations of the [002], [111], and [020] peaks are consistent with the previously reported rotator phase spectra. The *a*, *b*, and *c* lattice parameters computed from these peaks will be reported alongside the values obtained from the MD simulation.

**Table 1:** *q* Values of X-ray Diffraction Peaks for the C_16_ Rotator Phase (Orthorhombic R_I_) Determined from the Synchrotron Experiment

	*q* value of peak [Å^–1^]					
	[002]	[111]	[113]	[020]	[200]	[131]
Sirota and Herhold^13^ (≈289 K)	0.283^a^	1.498		1.632		
Ueno et al.^19^ (≈281 K)	0.280	1.50		1.65		
Shinohara et al.^20^ (286 K)	0.281	1.49		1.63		
this work (289 K)	0.2805	1.494	1.546	1.625	2.493	2.740

a *q* value is obtained graphically from Figure 2 of ref (13).

### Simulation Results

#### Computed Lattice Parameters

We begin with the triclinic (non-rotator) phase to test the MD prediction of unit cell dimensions for this well-characterized phase. Lattice parameters computed from MD simulation of the triclinic phase are given in Table 2 at temperatures 273 to 288 K. We observe that a triclinic crystal structure is stable when using the Williams 7B force field and that the triclinic cell angles α, β, and γ are in particularly good agreement with experimental data. The main deficiency of the model is that the *b* lattice dimension is approximately 0.2 Å smaller than the experimental data, a relative error of 4%. This can be partly explained by the fact that the Williams force field uses a reduced C–H bond length of 1.04 Å, decreasing the effective size of the molecule along the *b* lattice dimension and allowing the chains to pack more closely. Previously, Polson and Frenkel found that another version of the Williams force field could accurately reproduce the triclinic cell angles of *n*-octane, and they also reported an underestimation of the *b* lattice vector by ≈ 0.2 Å.^44^

**Table 2:** Lattice Parameters of the C_16_ Triclinic Phase, Comparing Experimental Data to Prediction from MD Simulation with the Williams 7B Force Field^a^

		*a* [Å]	*b* [Å]	*c* [Å]	α [°]	β [°]	γ [°]
	experiment^16^ (273 K)	4.269	4.811	22.345	84.54	67.43	73.00
this work	simulation (273 K)	4.328	4.613	22.510	84.47	67.77	74.30
	simulation (278 K)	4.336	4.618	22.513	84.46	67.75	74.17
	simulation (283 K)	4.343	4.623	22.516	84.45	67.72	74.05
	simulation (288 K)	4.352	4.629	22.520	84.43	67.69	73.90

a Uncertainties are provided in Supporting Information Table S3.

Another property closely related to the lattice parameters is the tilt angle, θ_tilt_, of the molecules with respect to the crystal *ab*-plane normal. This can be computed by determining the angle between the average  vector (see Figure 3a) and the *z* axis since the *z* axis is normal to the *ab*-plane in our system. Provided the average  vector is normalized, this is given by , where  is the *z* component of . Métivaud et al. determined θ_tilt_ = 19.07° for C_16_ at 273 K using X-ray powder diffraction.^16^ At the same temperature, averaged over all 1000 simulation frames, we obtain θ_tilt_ = 18.97 ± 0.02° and therefore good agreement for the tilt angle of the triclinic phase.

Computed lattice parameters of the orthorhombic rotator phase observed in the MD simulation are given in Table 3. Dimensions *a* and *b* are in somewhat better agreement than for the triclinic phase, being underestimated by 0.5 and 2.2%, respectively. We find the *b*/*a* ratio increases nonlinearly with temperature, which is the expected behavior of the R_I_ phase as observed for odd *n*-alkanes such as C_15_^12^ (noting that the directions of *a* and *b* are swapped in Ungar’s work). In the following sections, we provide more evidence that the MD simulated rotator phase is the face-centered orthorhombic R_I_ phase with the *Fmmm* space group.

**Table 3:** Lattice Parameters of the C_16_ R_I_ Rotator Phase^a^ Determined from X-ray Spectra and MD Simulation^b^

		*a* [Å]	*b* [Å]	*c* [Å]	*b*/*a*
this work	experiment (289 K)	5.043	7.735	44.795	1.534
	simulation (288 K)	5.017	7.561	44.779	1.507
	simulation (293 K)	5.006	7.620	44.800	1.522
	simulation (298 K)	4.986	7.707	44.834	1.546
	simulation (303 K)	4.913	7.942	44.938	1.617
Shinohara et al.^20^	experiment^c^ (286 K)	5.07	7.71	44.72	1.521
Cholakova et al.^14^	experiment^d^ (288 K)	5.03	7.70	44.74	1.530

a As this is an orthorhombic phase, α = β = γ = 90°.

b Uncertainties are provided in Supporting Information Table S4.

c Lattice parameters computed from the diffraction peaks in ref (20) making use of eq 6.

d Lattice parameters averaged using data in ref (14) provided by lead author D.C.

#### Positional Order

**Figure 7:**
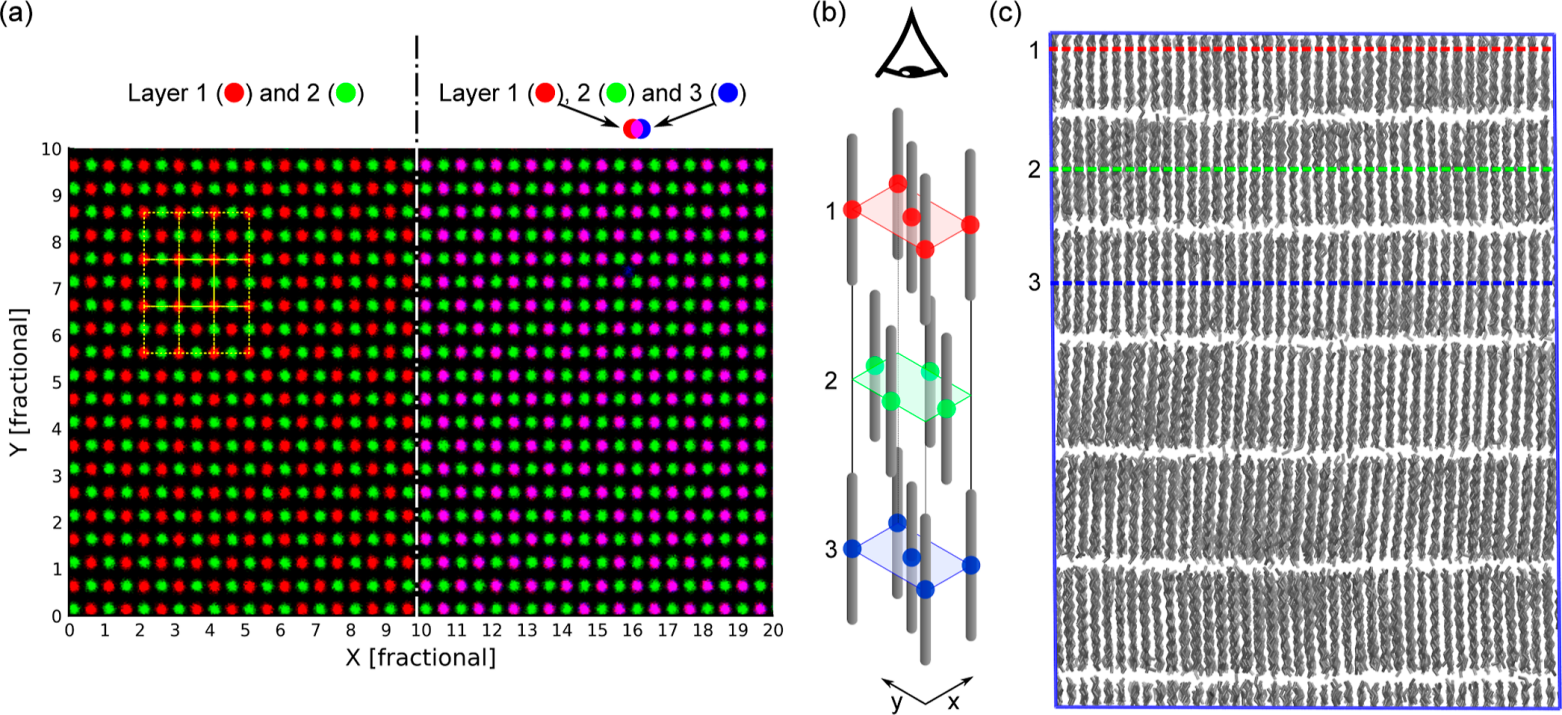
(a) Number density profiles of molecules in the first three layers of the C_16_ rotator phase simulation. The density of layers 1, 2, and 3 are assigned to the color channels red, green, and blue, respectively. Yellow rectangles indicate the base of the unit cell; (b) viewing direction and expected molecule positions of a face-centered orthorhombic cell; (c) three layers labeled on the MD simulation box.

In Figure 7 we visualize the average molecular positions during the MD simulation of the R_I_ phase and verify that they are consistent with a face-centered orthorhombic structure. This is done by creating a spatial density plot for each layer and assigning each of the three layers to a different color channel to create an image. In Figure 7a (left), this is done for the first two layers only, which are assigned to the red and green channels, respectively. From this, we see that the molecules in the second layer (colored green) lie at the mid-points of the faces of a rectangular unit cell, which is drawn as a yellow box in Figure 7a. Then, in Figure 7a (right), the density of the first three layers is assigned to red, green, and blue color channels, respectively. We observe that the molecular positions in the third layer coincide with those in the first layer, which results in the red and blue color channels overlapping to create magenta. This is consistent with a face-centered orthorhombic structure as shown in Figure 7b. We then proceed to analyze the molecular orientation (or distribution of orientations) to see if it is consistent with the R_I_ rotator phase.

#### Rotational Order

**Figure 8:**
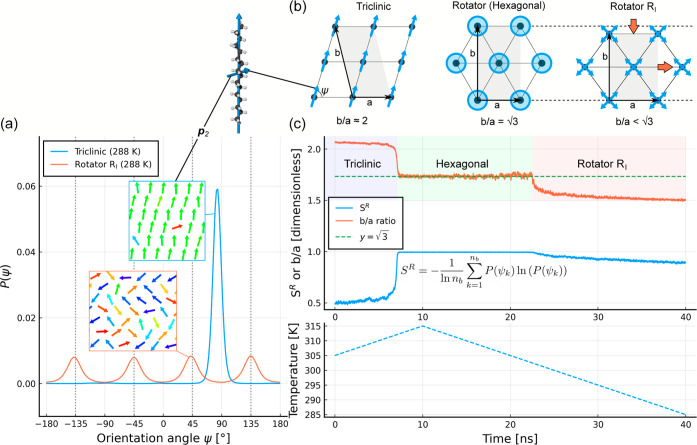
(a) Distribution of **p**_2_ orientation angles measured from constant temperature simulations of the triclinic and R_I_ phases; (b) structure of the three phases viewed in the *ab*-plane; (c) dimensionless *b*/*a* ratio and rotational entropy *S*^*R*^, computed during a heating and cooling simulation (temperature profile in the lower plot with a shared *x* axis) which undergoes triclinic to hexagonal to R_I_ phase transitions.

In the MD simulation, the transition from the triclinic phase to the R_I_ rotator phase occurs via an intermediate phase, which we will show is a hexagonal rotator phase. This is due to the high temperature used in the simulation to initiate the crystal-rotator transition. We analyze this process using the lattice parameters and the **p**_2_ orientation vectors (see Figure 3a) with their associated rotational entropy (eq 4). In Figure 8a, the distribution of **p**_2_ orientation angles is shown for the triclinic and R_I_ phases, as computed from the 15 °C simulations. The orientation angle ψ is defined as the angle between the **p**_2_ vector and the *a* axis.

In the triclinic phase, the vast majority of molecules share a common orientation angle, which is approximately 84°. In the R_I_ rotator phase, we observe four distinct peaks of equal height and area underneath, indicating that the **p**_2_ vector has four equally preferred orientations at ψ = ±45° and ± 135°. The distribution of gauche dihedral defects along the carbon backbone for the rotator phase is also provided in Supporting Information Figure S7.

As we must superheat the crystal to increase molecular mobility and allow the transition to the rotator phase, we observe an intermediate phase in which the *b*/*a* ratio is close to , indicating a hexagonal phase, which then transitions to the R_I_ phase upon cooling. Within this hexagonal phase, the rotational entropy is unity, which indicates a high level of rotational disorder and no significantly preferred orientations of the **p**_2_ vectors.

### Constructed CIF and Computed Spectra

CIFs for two potential orthorhombic unit cells of C_16_ were produced, both with the same unit cell dimensions (our experimental values in Table 3) but different space groups. The first has the *Fmmm* space group, which is characteristic of the R_I_ rotator phase. The second is provided for comparison purposes only and has the *Pca*2_1_ space group, which is the space group *n*-alkanes (*n* even) typically have if they form the analogous orthorhombic phase without the rotational disorder. *Pca*2_1_, however, is only the lowest energy structure for longer even n-alkanes, with C_36_ being the shortest to demonstrate it.^42,45^ In order to provide a direct comparison where the space group is the only variable, the unit cell dimensions of the hypothetical *Pca*2_1_ cell were set equal to the R_I_ cell. However, the base of the reported R_I_ unit cell is 5.04 × 7.74 Å (39.01 Å^2^), significantly larger than the base of the C_36_*Pca*2_1_ unit cell reported by Teare^45^ of 4.96 × 7.42 Å (36.80 Å^2^). In other words, the increased rotational freedom appears to increase the area per molecule in the *ab*-plane. It is therefore unlikely that the reported R_I_ phase is actually a non-rotator *Pca*2_1_ crystal. A visualization of this hypothetical *Pca*2_1_ unit cell is provided in Supporting Information Figure S8.

**Figure 9:**
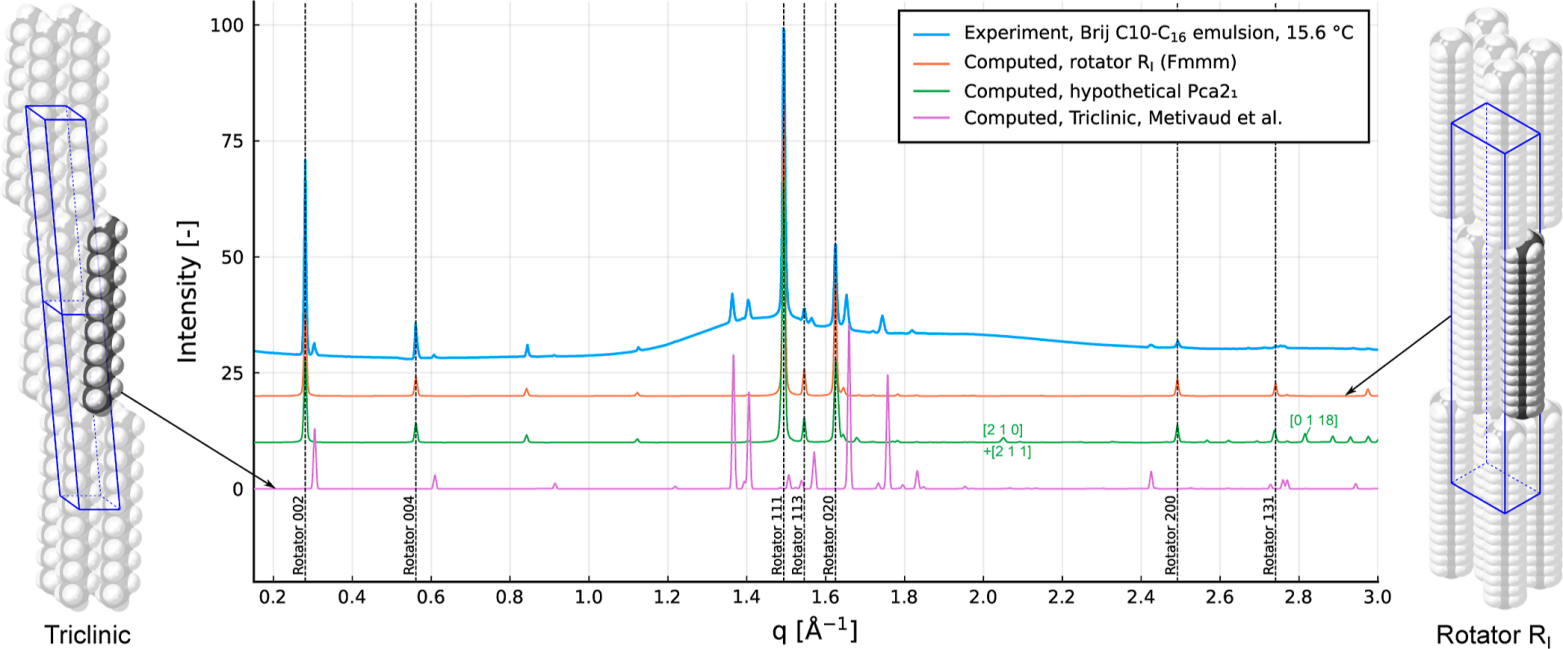
Comparison of synchrotron spectra to computed spectra for both the R_I_ and triclinic phases. The computed spectra are determined using CrystalDiffract^37^ from CIFs, with the CIF for the R_I_ phase being created in this work.

Figure 9 contains the powder diffraction spectra computed using CrystalDiffract^37^ from the CIFs of the two possible orthorhombic unit cells of C_16_ (*Fmmm* and *Pca*2_1_ space groups) as well as the spectra computed from the known triclinic unit cell,^16^ all alongside the experimentally measured spectra.

The computed spectra obtained for the orthorhombic cells are very similar since they have the same cell dimensions, but there are peaks present in the *Pca*2_1_ spectra which are systematically absent for the face-centered orthorhombic *Fmmm*. For a peak with reflection indices [*hkl*] to be present, the face-centered orthorhombic lattice requires the conditions *h* + *k* = 2*p*, *h* + *l* = 2*q*, and *k* + *l* = 2*r*, where *p*, *q*, and *r* are integers.^46^ In other words, the sum of any two peak indices must be even. This can only be satisfied if all indices are even or all indices are odd. In Figure 9, some peaks of the *Pca*2_1_ spectra are labeled which violate this condition, such as [2 1 0] and [0 1 18], which are therefore absent in the *Fmmm* spectra. As these peaks are not observed in the experimental spectra, this is further evidence that the C_16_ rotator phase is the R_I_ phase with the *Fmmm* space group.

The final CIF for the R_I_ rotator phase is provided in the Supporting Information archive. The four equally probable orientations we observe in the MD simulation are consistent with the mirror symmetry elements of the *Fmmm* space group and the fact that the molecules have four nearest-neighbors (≈4.6 Å away) on the face-centered orthorhombic lattice, but other arrangements may be possible. As the orientation of the molecule in the asymmetric unit is derived from the MD simulations, this is one aspect of the unit cell that could warrant further refinement. Importantly, the unit cell dimensions in the provided CIF are based on the spectra peak locations and therefore not dependent on the accuracy of the MD results.

Comparing the experimental and computed spectra for the R_I_ phase in Figure 9, we obtain a good level of agreement in both peak locations and intensities. The computed diffraction pattern correctly predicts that the [002], [111], and [020] peaks are the most intense, followed by [004] and [113], then [200], and faintly [131]. Using Figure 9, we can also identify which peaks belong to the triclinic phase that is also present in the droplets.

## Conclusions

Synchrotron SAXS diffraction spectra showed a sequence of peaks [002], [004], [006], etc., that demonstrate the characteristic layer *d*-spacing of the orthorhombic R_I_ rotator phase of C_16_. Identification of two further peaks, corresponding to the [111] and [020] reflections, allowed determination of the crystal unit cell dimensions. Simulations of the rotator phase using the Williams 7B force field were performed to provide additional information as to the structure of this phase, which demonstrated that the C_16_ molecules occupied four rotational positions, which are equally spaced by almost exactly 90°. Combining the orientations predicted by the MD simulation and the inferred unit cell parameters from the X-ray spectra, we obtained the structure of the R_I_ phase with the *Fmmm* space group. A full unit cell model was created in the CIF format in which the molecule is represented as a superposition of the four possible orientations, each with 25% occupancy.

Using the generated CIF, the full powder diffraction spectra were computed using the CrystalDiffract^37^ software. Comparing these spectra to the X-ray diffraction pattern showed excellent agreement in both the location and intensity of the peaks that were observed. The R_I_ phase [113], [200], and [131] peaks were also detected, which were not identified in previous studies. X-ray spectra simulated using a hypothetical unit cell without the rotational disorder produced both the observed peaks and additional ones not observed in the experiments.

Since we are now able to generate the complete spectra of the rotator phase, it is possible to locate all relevant peaks and therefore easier to process complex diffraction spectra with multiple phases present. The methodology used here for creating the R_I_ phase CIF and generating X-ray spectra from it can be used to identify and characterize other rotator phases.

## Supporting Information Available

Visualization of unit cells, X-ray peak fitting, dihedral distribution, force field parameters, and MD result uncertainties (PDF)
*n*-Hexadecane R_I_ rotator phase CIF and Williams 7B force field files in the Moltemplate format (for LAMMPS) (ZIP)

## References

[ref1] Denkov N.; Tcholakova S.; Lesov I.; Cholakova D.; Smoukov S. K. Self-shaping of oil droplets via the formation of intermediate rotator phases upon cooling. Nature 2015, 528, 392–395. 10.1038/nature16189. 26649824

[ref2] Denkov N.; Cholakova D.; Tcholakova S.; Smoukov S. K. On the mechanism of drop self-shaping in cooled emulsions. Langmuir 2016, 32, 7985–7991. 10.1021/acs.langmuir.6b01626. 27429158

[ref3] Liber S. R.; Marin O.; Butenko A. V.; Ron R.; Shool L.; Salomon A.; Deutsch M.; Sloutskin E. Polyhedral water droplets: shape transitions and mechanism. J. Am. Chem. Soc. 2020, 142, 8672–8678. 10.1021/jacs.0c00184. 32307985

[ref4] García-Aguilar I.; Fonda P.; Sloutskin E.; Giomi L. Faceting and flattening of emulsion droplets: A mechanical model. Phys. Rev. Lett. 2021, 126, 038001 10.1103/physrevlett.126.038001. 33543952

[ref5] Haas P. A.; Goldstein R. E.; Cholakova D.; Denkov N.; Smoukov S. K. Comment on “Faceting and Flattening of Emulsion Droplets: A Mechanical Model”. Phys. Rev. Lett. 2021, 126, 259801 10.1103/physrevlett.126.259801. 34241513

[ref6] Haas P. A.; Goldstein R. E.; Smoukov S. K.; Cholakova D.; Denkov N. Theory of shape-shifting droplets. Phys. Rev. Lett. 2017, 118, 088001 10.1103/physrevlett.118.088001. 28282177

[ref7] Haas P. A.; Cholakova D.; Denkov N.; Goldstein R. E.; Smoukov S. K. Shape-shifting polyhedral droplets. Phys. Rev. Res. 2019, 1, 023017 10.1103/physrevresearch.1.023017. 28282177

[ref8] Tcholakova S.; Valkova Z.; Cholakova D.; Vinarov Z.; Lesov I.; Denkov N.; Smoukov S. K. Efficient self-emulsification via cooling-heating cycles. Nat. Commun. 2017, 8, 15012 10.1038/ncomms15012. 28447603 PMC5457670

[ref9] Cholakova D.; Glushkova D.; Tcholakova S.; Denkov N. Cold-burst method for nanoparticle formation with natural triglyceride oils. Langmuir 2021, 37, 7875–7889. 10.1021/acs.langmuir.0c02967. 33586441

[ref10] Lesov I.; Valkova Z.; Vassileva E.; Georgiev G. S.; Ruseva K.; Simeonov M.; Tcholakova S.; Denkov N. D.; Smoukov S. K. Bottom-up synthesis of polymeric micro-and nanoparticles with regular anisotropic shapes. Macromolecules 2018, 51, 7456–7462. 10.1021/acs.macromol.8b00529.

[ref11] Cholakova D.; Denkov N.; Tcholakova S.; Lesov I.; Smoukov S. K. Control of drop shape transformations in cooled emulsions. Adv. Colloid Interface Sci. 2016, 235, 90–107. 10.1016/j.cis.2016.06.002. 27389390

[ref12] Ungar G. Structure of rotator phases in n-alkanes. J. Phys. Chem. 1983, 87, 689–695. 10.1021/j100227a032.

[ref13] Sirota E.; Herhold A. Transient rotator phase induced nucleation in n-alkane melts. Polymer 2000, 41, 8781–8789. 10.1016/s0032-3861(00)00221-4.

[ref14] Cholakova D.; Glushkova D.; Valkova Z.; Tsibranska-Gyoreva S.; Tsvetkova K.; Tcholakova S.; Denkov N. Rotator phases in hexadecane emulsion drops revealed by X-ray synchrotron techniques. J. Colloid Interface Sci. 2021, 604, 260–271. 10.1016/j.jcis.2021.06.122. 34271488

[ref15] Cholakova D.; Denkov N.; Tcholakova S.; Valkova Z.; Smoukov S. K. Multilayer formation in self-shaping emulsion droplets. Langmuir 2019, 35, 5484–5495. 10.1021/acs.langmuir.8b02771. 30924339

[ref16] Métivaud V.; Lefèvre A.; Ventolà L.; Négrier P.; Moreno E.; Calvet T.; Mondieig D.; Cuevas-Diarte M. A. Hexadecane (C_16_H_34_)+ 1-hexadecanol (C_16_H_33_OH) binary system: crystal structures of the components and experimental phase diagram. Application to thermal protection of liquids. Chem. Mater. 2005, 17, 3302–3310. 10.1021/cm050130c.

[ref17] Groom C. R.; Bruno I. J.; Lightfoot M. P.; Ward S. C. The Cambridge structural database. Acta Crystallogr., Sect. B: Struct. Sci., Cryst. Eng. Mater. 2016, 72, 171–179. 10.1107/s2052520616003954. PMC4822653 27048719

[ref18] Di Giambattista C.; Sanctuary R.; Perigo E.; Baller J. Relaxations in the metastable rotator phase of n-eicosane. J. Chem. Phys. 2015, 143, 054507 10.1063/1.4928059. 26254661

[ref19] Ueno S.; Hamada Y.; Sato K. Controlling polymorphic crystallization of n-alkane crystals in emulsion droplets through interfacial heterogeneous nucleation. Cryst. Growth Des. 2003, 3, 935–939. 10.1021/cg0300230.

[ref20] Shinohara Y.; Kawasaki N.; Ueno S.; Kobayashi I.; Nakajima M.; Amemiya Y. Observation of the transient rotator phase of n-hexadecane in emulsified droplets with time-resolved two-dimensional small-and wide-angle X-ray scattering. Phys. Rev. Lett. 2005, 94, 097801 10.1103/physrevlett.94.097801. 15784000

[ref21] Cockcroft J. K.; Shamsabadi A.; Wu H.; Rennie A. R. Understanding the structure and dynamics of cationic surfactants from studies of pure solid phases. Phys. Chem. Chem. Phys. 2019, 21, 25945–25951. 10.1039/c9cp04486h. 31595275

[ref22] Falls A.; Scriven L.; Davis H. Adsorption, structure, and stress in binary interfaces. J. Chem. Phys. 1983, 78, 7300–7317. 10.1063/1.444720.

[ref23] Stephan S.; Hasse H. Interfacial properties of binary mixtures of simple fluids and their relation to the phase diagram. Phys. Chem. Chem. Phys. 2020, 22, 12544–12564. 10.1039/d0cp01411g. 32452484

[ref24] da Silva G. C.; Silva G. M.; Tavares F. W.; Fleming F. P.; Horta B. A. Are all-atom any better than united-atom force fields for the description of liquid properties of alkanes?. J. Mol. Model. 2020, 26, 296 10.1007/s00894-020-04548-5. 33026509

[ref25] Schmitt S.; Fleckenstein F.; Hasse H.; Stephan S. Comparison of Force Fields for the Prediction of Thermophysical Properties of Long Linear and Branched Alkanes. J. Phys. Chem. B 2023, 127, 1789–1802. 10.1021/acs.jpcb.2c07997. 36802607

[ref26] Glova A. D.; Volgin I. V.; Nazarychev V. M.; Larin S. V.; Lyulin S. V.; Gurtovenko A. A. Toward realistic computer modeling of paraffin-based composite materials: Critical assessment of atomic-scale models of paraffins. RSC Adv. 2019, 9, 38834–38847. 10.1039/c9ra07325f. 35540183 PMC9076000

[ref27] Burrows S. A.; Korotkin I.; Smoukov S. K.; Boek E.; Karabasov S. Benchmarking of molecular dynamics force fields for solid–liquid and solid–solid phase transitions in alkanes. J. Phys. Chem. B 2021, 125, 5145–5159. 10.1021/acs.jpcb.0c07587. 33724846

[ref28] Ryckaert J.-P.; Klein M. L. Translational and rotational disorder in solid n-alkanes: Constant temperature–constant pressure molecular dynamics calculations using infinitely long flexible chains. J. Chem. Phys. 1986, 85, 1613–1620. 10.1063/1.451203.

[ref29] Marbeuf A.; Brown R. Molecular dynamics in n-alkanes: Premelting phenomena and rotator phases. J. Chem. Phys. 2006, 124, 054901 10.1063/1.2148909. 16468912

[ref30] Sun H. COMPASS: An ab Initio Force-Field Optimized for Condensed-Phase ApplicationsOverview with Details on Alkane and Benzene Compounds. J. Phys. Chem. B 1998, 102, 7338–7364. 10.1021/jp980939v.

[ref31] Wentzel N.; Milner S. T. Crystal and rotator phases of n-alkanes: A molecular dynamics study. J. Chem. Phys. 2010, 132, 044901 10.1063/1.3276458. 20113060

[ref32] Wentzel N.; Milner S. T. Simulation of multiple ordered phases in C_23_ n-alkane. J. Chem. Phys. 2011, 134, 224504 10.1063/1.3589417. 21682522

[ref33] Williams D. E. Nonbonded potential parameters derived from crystalline hydrocarbons. J. Chem. Phys. 1967, 47, 4680–4684. 10.1063/1.1701684.

[ref34] Iliev S.; Tsibranska S.; Ivanova A.; Tcholakova S.; Denkov N. Computational assessment of hexadecane freezing by equilibrium atomistic molecular dynamics simulations. J. Colloid Interface Sci. 2023, 638, 743–757. 10.1016/j.jcis.2023.01.126. 36780853

[ref35] Thureau A.; Roblin P.; Pérez J. BioSAXS on the SWING beamline at Synchrotron SOLEIL. J. Appl. Crystallogr. 2021, 54, 1698–1710. 10.1107/s1600576721008736.

[ref36] Bezanson J.; Edelman A.; Karpinski S.; Shah V. B. J. Julia: A Fresh Approach to Numerical Computing. SIAM Rev. 2017, 59, 65–98. 10.1137/141000671.

[ref37] Palmer D.; Palmer S. CrystalMaker, v 10.6.0, 2021. www.crystalmaker.com (accessed Jul 03, 2023).

[ref38] Thompson A. P.; Aktulga H. M.; Berger R.; Bolintineanu D. S.; Brown W. M.; Crozier P. S.; in’t Veld P. J.; Kohlmeyer A.; Moore S. G.; Nguyen T. D.; et al. LAMMPS-a flexible simulation tool for particle-based materials modeling at the atomic, meso, and continuum scales. Comput. Phys. Commun. 2022, 271, 108171 10.1016/j.cpc.2021.108171.

[ref39] Jewett A. I.; Stelter D.; Lambert J.; Saladi S. M.; Roscioni O. M.; Ricci M.; Autin L.; Maritan M.; Bashusqeh S. M.; Keyes T.; et al. Moltemplate: A tool for coarse-grained modeling of complex biological matter and soft condensed matter physics. J. Mol. Biol. 2021, 433, 166841 10.1016/j.jmb.2021.166841. 33539886 PMC8119336

[ref40] Parrinello M.; Rahman A. Polymorphic transitions in single crystals: A new molecular dynamics method. J. Appl. Phys. 1981, 52, 7182–7190. 10.1063/1.328693.

[ref41] Smith A. The crystal structure of the normal paraffin hydrocarbons. J. Chem. Phys. 1953, 21, 2229–2231. 10.1063/1.1698826.

[ref42] Craig S. R.; Hastie G. P.; Roberts K. J.; Sherwood J. N. Investigation into the structures of some normal alkanes within the homologous series C_13_H_28_ to C_60_H_122_ using high-resolution synchrotron X-ray powder diffraction. J. Mater. Chem. 1994, 4, 977–981. 10.1039/jm9940400977.

[ref43] Maroncelli M.; Qi S. P.; Strauss H. L.; Snyder R. G. Nonplanar conformers and the phase behavior of solid n-alkanes. J. Am. Chem. Soc. 1982, 104, 6237–6247. 10.1021/ja00387a013.

[ref44] Polson J. M.; Frenkel D. Numerical prediction of the melting curve of n-octane. J. Chem. Phys. 1999, 111, 1501–1510. 10.1063/1.479409.

[ref45] Teare P. The crystal structure of orthorhombic hexatriacontane, C_36_H_74_. Acta Crystallogr. 1959, 12, 294–300. 10.1107/s0365110x59000901.

[ref46] Aroyo M. I.; Flack H.; Shmueli U. International Tables for Crystallography Volume A; Wiley Online Library, 2016. Chapter 1.6.4.

