# Supplementary material for: Structure of the Hexadecane Rotator Phase: Combination of X-ray Spectra and Molecular Dynamics Simulation
Source: J Phys Chem B. 2023 Aug 30;127(36):7772–84. doi: 10.1021/acs.jpcb.3c02027 (PMC10510391; doi:10.1021/acs.jpcb.3c02027)
Supplement: Supplementary file 1 — jp3c02027_si_001.pdf [file jp3c02027_si_001.pdf]

Supporting Information: Structure of the Hexadecane Rotator Phase: Combination of X-ray Spectra and Molecular Dynamics Simulation

Stephen A. Burrows,[†] E Emily Lin,[†] Diana Cholakova,[‡] Sam Richardson,[†,¶] and Stoyan K. Smoukov[*,†]

†*Centre for Sustainable Engineering, School of Engineering and Materials Science, Queen Mary University of London, Mile End Road, London, E1 4NS, UK*

‡*Department of Chemical and Pharmaceutical Engineering, Faculty of Chemistry and Pharmacy, Sofia University, 1164 Sofia, Bulgaria*

¶*Present affiliation: Imaging and Data Analytics, Clinical Pharmacology & Safety Sciences, R&D, AstraZeneca, Cambridge, CB2 0AA, UK*

E-mail: s.smoukov@qmul.ac.uk

Phone: +44 (0)20 7882 5305

Supporting Information Available

Transformation of Triclinic Unit Cell

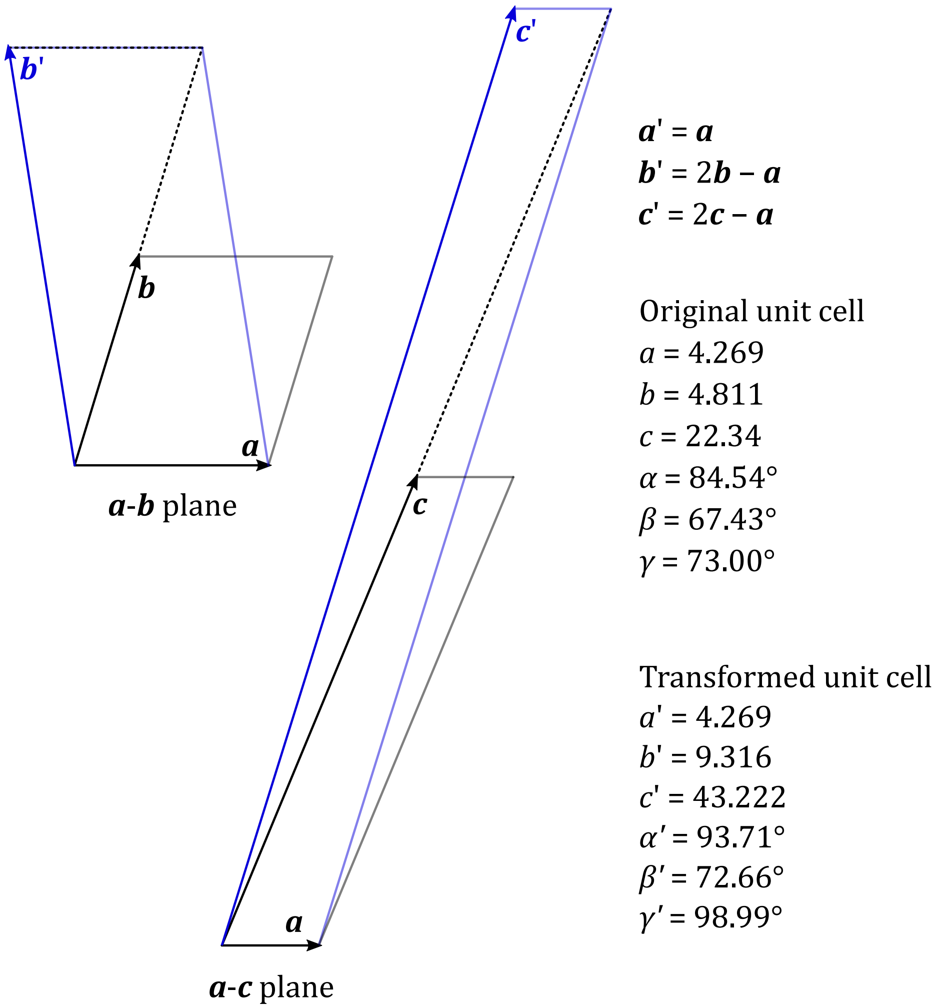

Figure S1: Transformations applied to primitive triclinic lattice vectors ($\mathbf{a}$, $\mathbf{b}$, $\mathbf{c}$) to obtain non-primitive unit cell with lattice vectors ($\mathbf{a}'$, $\mathbf{b}'$, $\mathbf{c}'$).

Williams 7B Force Field Parameters

Table S1: Buckingham potential parameters of the Williams 7B force field*

Version	Interaction	A [kJ mol^{-1}]	B [nm^{-1}]	C [nm^6 kJ mol^{-1}]
	C-C	341.86×10^3	36.94	2.005×10^{-3}
Williams 7B	C-H	58.14×10^3	37.61	0.507×10^{-3}
	H-H	13.65×10^3	38.30	0.128×10^{-3}

*Parameters are given for the Buckingham potential form $U_{\text{Buck}}(r) = A \exp(-Br) - C/r^6$. Intramolecular non-bonded interactions are excluded for atoms separated by three bonds or less.

Table S2: Bonding potential parameters of the Williams 7B force field

	Bond†, $U_b(b) = \frac{1}{2}k_b(b-b_0)^2$	
	b_0 [nm]	k_b [kJ mol^{-1} nm^{-2}]
C-C	0.153	224262†
C-H	0.104	284512†
	Angle†, $U_\theta(\theta) = \frac{1}{2}k_\theta(\theta-\theta_0)^2$	
	θ_0 [°]	k_θ [kJ mol^{-1} rad^{-2}]
C-C-C	113.7	488.273†
C-C-H	109.2	313.800†
H-C-H	106.0	276.144†
	Torsion potential $U_\phi = \sum_n k_n(1+\cos(n\phi))$	
	n	k_n [kJ mol^{-1}]
C-C-C-C	1	0.9791
	2	0.4017
	3	1.7154
C-C-C-H	3	0.7406
H-C-C-H	3	0.6904

†GROMACS includes the prefactor of $\frac{1}{2}$ in the harmonic potentials whereas LAMMPS does not. The supporting archive contains the same parameters in LAMMPS units.

X-ray Peak Fitting

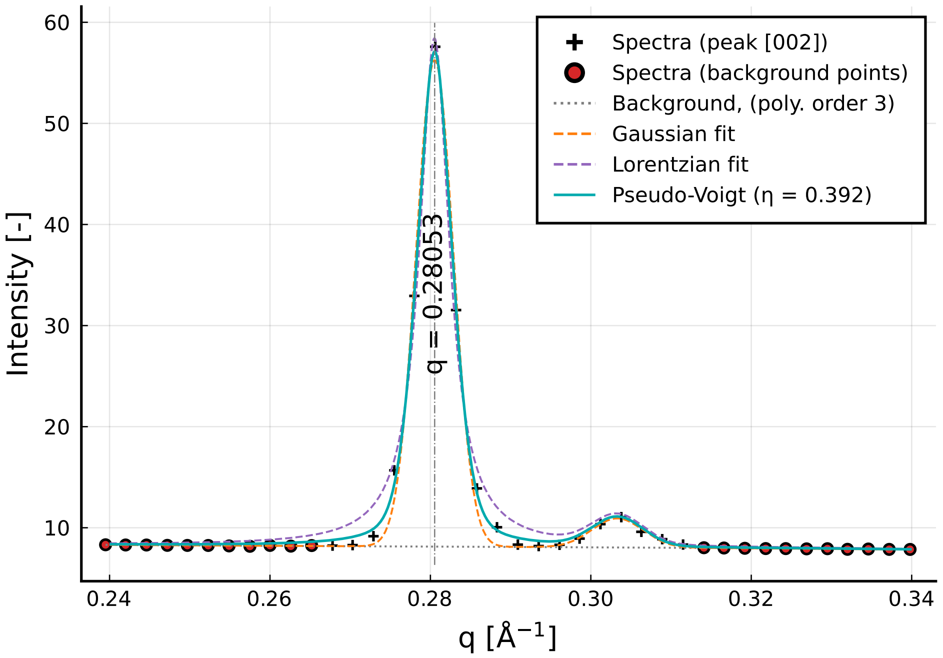

Figure S2: Fitting of [002] rotator phase diffraction peak measured from Brij C10 stabilized C_{16} emulsion at 15.6 °C.

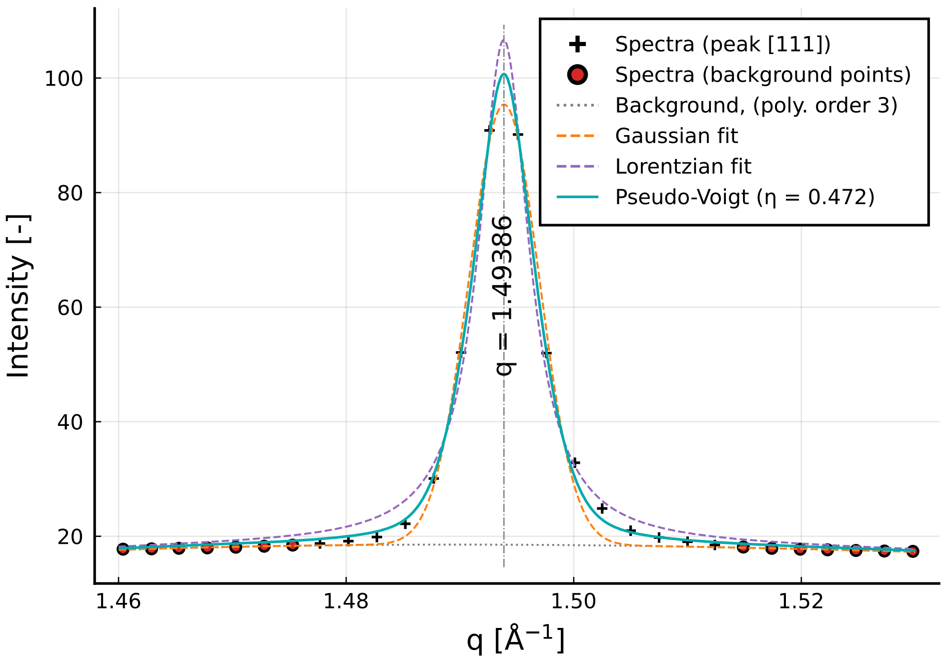

Figure S3: Fitting of [111] rotator phase diffraction peak measured from Brij C10 stabilized C_{16} emulsion at 15.6 °C.

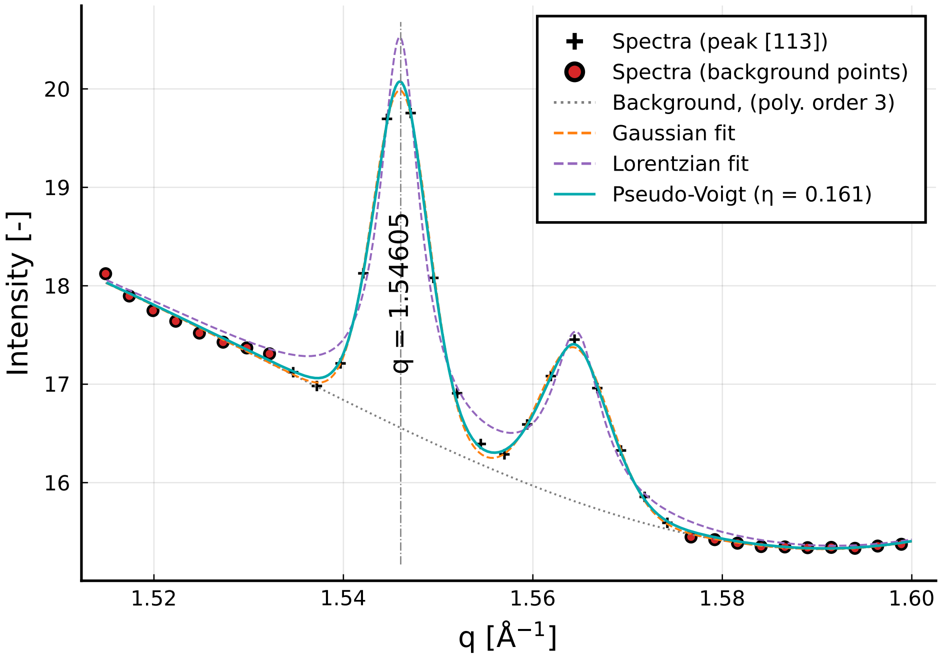

Figure S4: Fitting of [113] rotator phase diffraction peak measured from Brij C10 stabilized C_{16} emulsion at 15.6 °C.

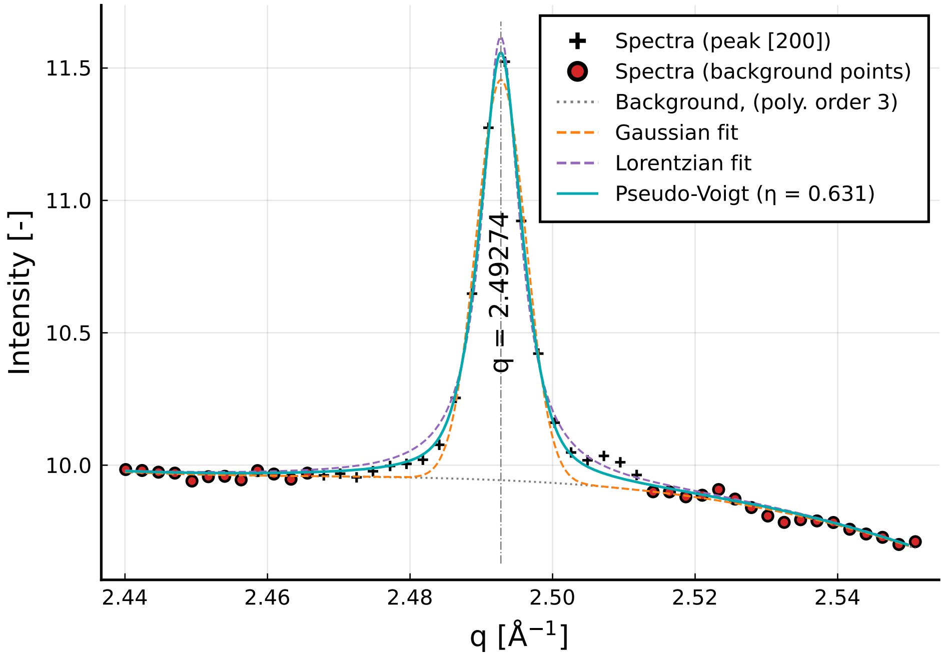

Figure S5: Fitting of [200] rotator phase diffraction peak measured from Brij C10 stabilized C_{16} emulsion at 15.6 °C.

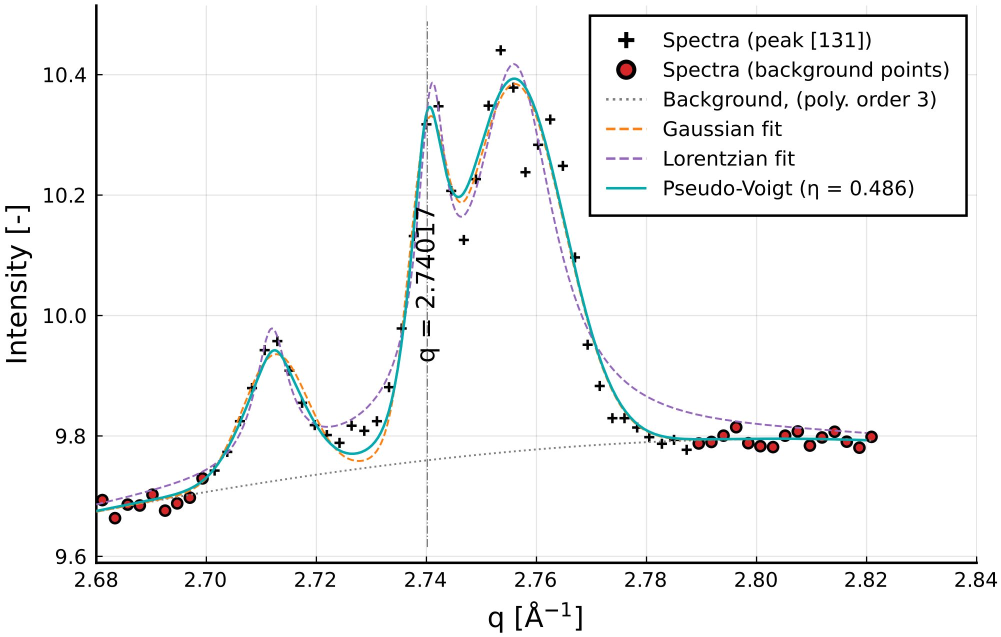

Figure S6: Fitting of [131] rotator phase diffraction peak measured from Brij C10 stabilized C_{16} emulsion at 15.6 °C. Based on the lattice parameters, the expected value of this peak is q = 2.7406 Å^{-1}, allowing it to be differentiated from the neighboring peaks.

Dihedral Distribution

Figure S7 contains the fraction of trans (anti periplanar) and gauche bonds in the rotator phase for the thirteen C-C-C-C dihedrals along the hexadecane chain. If the dihedral angle, ϕ, is in the range $120° < \phi < 240°$ the dihedral is categorized as trans, and otherwise it is gauche.

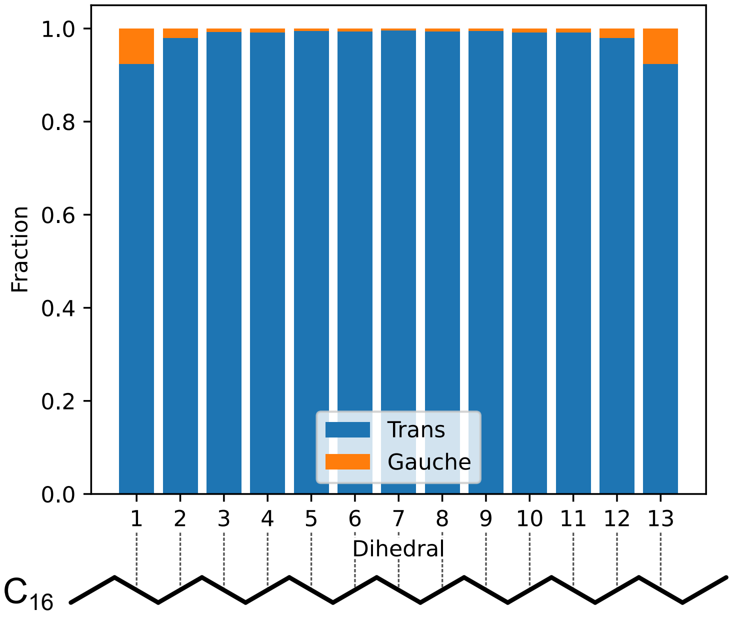

Figure S7: Distribution of trans and gauche dihedral angles along C_{16} backbone, computed from MD simulation of R_I phase at 293 K.

Hypothetical Pca2_1 Unit Cell

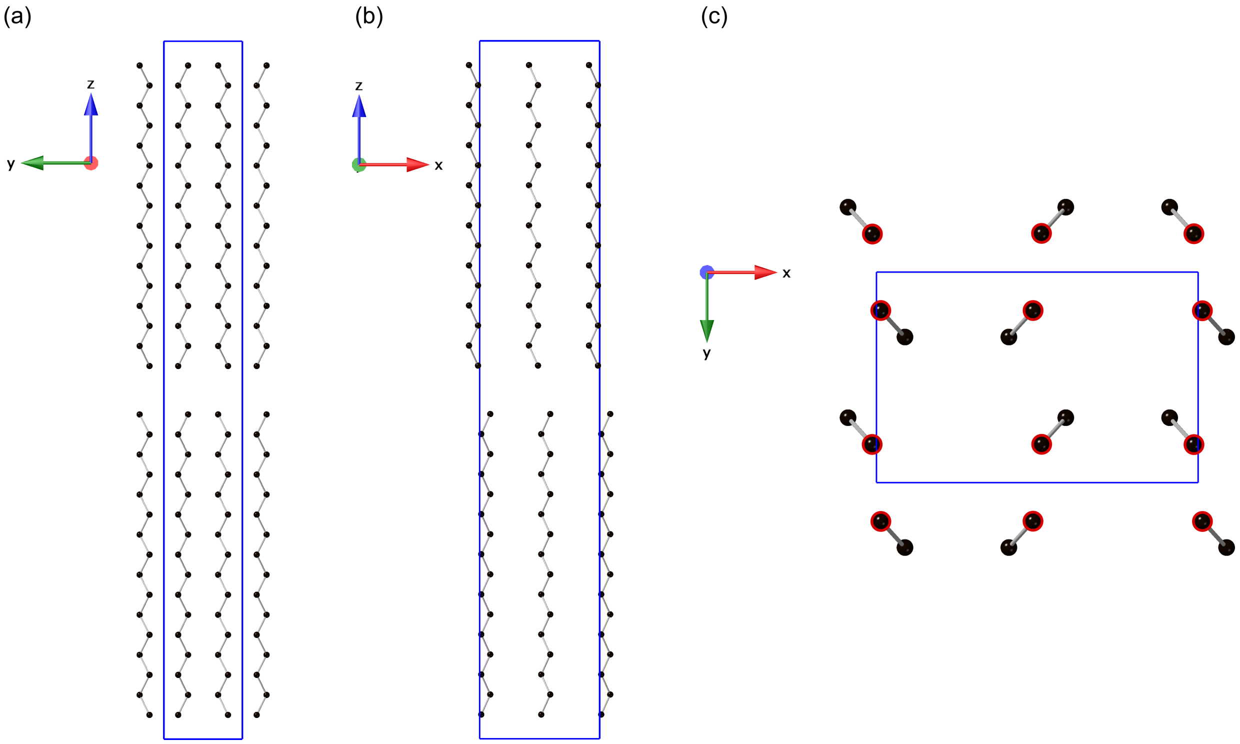

Figure S8: Visualization of hypothetical Pca2_1 unit cell, with hydrogens omitted for clarity. (a) view along a lattice direction; (b) view along b lattice direction; (c) view along c lattice direction. In (c), atoms closest to the viewer (C in terminal methyl groups) are outlined in red.

Computed Lattice Parameters with Uncertainties

Uncertainties in Table S3–4 are computed using standard deviations from a block averaging process. Each simulation is subdivided into 1 ns blocks and lattice parameters computed for each block. Then the standard deviation of these individual measurements is computed to estimate the uncertainty.

Table S3: Lattice parameters of C_{16} triclinic phase, comparing experimental data to prediction from MD simulation with the Williams 7B force field, with uncertainties given underneath each value

		a [Å]	b [Å]	c [Å]	α	β	γ
	Experiment[S1] (273 K)	4.269	4.811	22.345	84.54	67.43	73.00
This work:	Simulation (273 K)	4.3279	4.6128	22.5097	84.471	67.766	74.297
		±0.0003	±0.0002	±0.0005	±0.002	±0.003	±0.006
	Simulation (278 K)	4.3355	4.6182	22.5135	84.457	67.746	74.169
		±0.0006	±0.0003	±0.0009	±0.002	±0.003	±0.009
	Simulation (283 K)	4.3431	4.6234	22.5161	84.447	67.723	74.046
		±0.0005	±0.0003	±0.0006	±0.002	±0.003	±0.007
	Simulation (288 K)	4.3521	4.6293	22.5203	84.434	67.695	73.897
		±0.0008	±0.0004	±0.0009	±0.002	±0.003	±0.014

Table S4: Lattice parameters of R_I rotator phase[a] determined from X-ray spectra and MD simulation, with uncertainties given underneath each value

		a [Å]	b [Å]	c [Å]	b/a
This work:	Experiment (288 K)	5.043	7.735	44.795	1.534
	Simulation (288 K)	5.0166	7.5609	44.7791	1.5072
		±0.0006	±0.0036	±0.0059	±0.0009
	Simulation (293 K)	5.0057	7.6197	44.7995	1.5222
		±0.0007	±0.0037	±0.0066	±0.0009
	Simulation (298 K)	4.9858	7.7073	44.8341	1.5459
		±0.0013	±0.0069	±0.0075	±0.0018
	Simulation (303 K)	4.9130	7.9419	44.9384	1.6165
		±0.0138	±0.0382	±0.0220	±0.0123

[a]As this is an orthorhombic phase, $\alpha = \beta = \gamma = 90°$

References

(S1) Métivaud, V.; Lefèvre, A.; Ventolà, L.; Négrier, P.; Moreno, E.; Calvet, T.; Mondieig, D.; Cuevas-Diarte, M. A. Hexadecane (C16H34)+ 1-hexadecanol (C16H33OH) binary system: crystal structures of the components and experimental phase diagram. Application to thermal protection of liquids. *Chemistry of Materials* **2005**, *17*, 3302–3310.
